# *DCET1* Controls Male Sterility Through Callose Regulation, Exine Formation, and Tapetal Programmed Cell Death in Rice

**DOI:** 10.3389/fgene.2021.790789

**Published:** 2021-11-24

**Authors:** Riaz Muhammad Khan, Ping Yu, Lianping Sun, Adil Abbas, Liaqat Shah, Xiaojiao Xiang, Dongfei Wang, Amir Sohail, Yingxin Zhang, Qunen Liu, Shihua Cheng, Liyong Cao

**Affiliations:** ^1^ Key Laboratory for Zhejiang Super Rice Research and State Key Laboratory of Rice Biology, China National Rice Research Institute, Hangzhou, China; ^2^ Department of Botany, Mir Chakar Khan Rind University, Sibi, Pakistan

**Keywords:** male sterility, callose, pollen exine, tapetum, PCD, *DCET1*

## Abstract

In angiosperms, anther development comprises of various complex and interrelated biological processes, critically needed for pollen viability. The transitory callose layer serves to separate the meiocytes. It helps in primexine formation, while the timely degradation of tapetal cells is essential for the timely callose wall dissolution and pollen wall formation by providing nutrients for pollen growth. In rice, many genes have been reported and functionally characterized that are involved in callose regulation and pollen wall patterning, including timely programmed cell death (PCD) of the tapetum, but the mechanism of pollen development largely remains ambiguous. We identified and functionally characterized a rice mutant *dcet1*, having a complete male-sterile phenotype caused by defects in anther callose wall, exine patterning, and tapetal PCD. *DCET1* belongs to the RNA recognition motif (RRM)-containing family also called as the ribonucleoprotein (RNP) domain or RNA-binding domain (RBD) protein, having single-nucleotide polymorphism (SNP) substitution from G (threonine-192) to A (isoleucine-192) located at the fifth exon of LOC_Os08g02330, was responsible for the male sterile phenotype in mutant *dcet1*. Our cytological analysis suggested that *DCET1* regulates callose biosynthesis and degradation, pollen exine formation by affecting exine wall patterning, including abnormal nexine, collapsed bacula, and irregular tectum, and timely PCD by delaying the tapetal cell degeneration. As a result, the microspore of *dcet1* was swollen and abnormally bursted and even collapsed within the anther locule characterizing complete male sterility. GUS and qRT-PCR analysis indicated that *DCET1* is specifically expressed in the anther till the developmental stage 9, consistent with the observed phenotype. The characterization of *DCET1* in callose regulation, pollen wall patterning, and tapetal cell PCD strengthens our knowledge for knowing the regulatory pathways involved in rice male reproductive development and has future prospects in hybrid rice breeding.

## Introduction

Rice (*Oryza sativa* L.) is a staple food crop worldwide, feeding around three billion people, nearly half of the global population (Cheng et al., 2007). The global population is intensively increasing, and water resources and agricultural land for rice production are shrinking because of urban expansion and climatic changes. Therefore, new breeding strategies are critically needed to vertically enhance the rice production. Several measures can help overcome food shortage, such as eradicating soil problems, improving cultural practices, proper control of pests and diseases, use of fertilizers, proper utilization of water resources, and use of elite varieties and hybrids (Li et al., 2009). Hybrid rice production is one of the key technologies, specifically among the genetic options, to overcome the growing population’s food shortage. Rice is a self-pollinated crop; therefore, the male sterility technique is used to develop commercial hybrid parental lines (Chang et al., 2016). Male sterility produces infertile pollens, so that rice spikelets are incapable of setting seeds through self-pollination (Chang et al., 2016). Thus, pollen viability is an important character for improved rice production (Zou et al., 2017b). Pollen is the key source for improved rice grain production; therefore, understanding the mechanism of pollen development is extremely important for rice breeding (Zhang et al., 2011).

At the end of meiosis-II, the callose is degraded by the tapetum-secreted callases/glucanases, which initiate pollen wall formation (Shi et al., 2015). The first fabricated complex structure of the pollen wall is cellulose-formed primexine, which is the functional site for sporopollenin precursors (Jiang et al., 2013; Lou et al., 2014b; Zhang and Li, 2014). In higher plants, the pollen wall is a bilayer, and consists of an outer layer exine and an inner layer intine (Hu et al., 2014). The exine is the outer protective wall of pollen grains, which plays an important role in the selective flow of fluids across the pollen and attracting pollinators (Scott, 1994; Scott et al., 2004; Ariizumi and Toriyama, 2011). The pollen wall also protects the pollen from microbial attacks and various environmental stresses (Lou et al., 2014a).

In rice, pollen exine is majorly made up of sporopollenin. It consists of an outer layer of sexine (tectum), a foot layer (nexine), the middle bacula, and the tryphine in the cavities (Blackmore et al., 2007). The sexine usually identifies the species, while the nexine is used as a frame for exine formation (Shi et al., 2015). The exine (cell wall polymer), is involved in sperm protection, consisting of three major developmental processes, such as primexine, callose wall, and sporopollenin formation. The callose wall is paramount for pollen development, consists of β-1,3-linked glucose residues, and is first synthesized by pollen mother cells (PMCs) during meiosis initiation (Xie et al., 2012; Shi et al., 2016). The perimetric callose wall controls the integration of microspores and tetrad rupture during microsporogenesis (Richmond and Somerville, 2001; Verma and Hong, 2001; Nishikawa et al., 2005). In Arabidopsis, several callose synthase (CalS) or glucan synthase–like (GSL) genes, namely, *CalS5* (*GSL2*), *CalS7* (*GSL7*), *CalS9* (*GSL10*), *CalS10* (*GSL8*), *CalS11* (*GSL1*), and *CalS12* (*GSL5*) are reported for the regulation of different biological processes and adoption of stressed conditions (Xie et al., 2012). Among them, *CalS5* is specifically involved in the synthesis and functioning of the surrounding callose wall of the pollen (Dong et al., 2005; Nishikawa et al., 2005).

The four lobes of rice anther are attached with the same median of vascular and connective tissues. Each lobe has central microsporocytes and is surrounded by four layers of somatic cells, such as the outer epidermis, endothecium, middle layer, and innermost tapetum (Sun et al., 2018). The tapetal cell layer is adjacent to the anther locule, and provides essential nutrients for the microspores and secretes enzymes, such as β-1,3-glucanase (callase), which disintegrate the callose wall to release the young haploid microspores (Piffanelli et al., 1998; Li et al., 2006). Tapetum development and degeneration are well-organized processes, and disturbance usually leads to male sterility (Parish and Li, 2010). Timely tapetal cell programmed cell death (PCD) is thought to be the main stimulant for tapetum degeneration. Tapetal PCD is also associated with the degradation of callose and the formation of primexine on the microspore’s surface, which is the first step in pollen cell wall formation (Ariizumi and Toriyama, 2011). In contrast to animals, during the plant reproduction process, PCD is relatively more beneficial for cell breakage and release of un-useful cellular constituents into useful functional components of the cell (Wu and Cheung, 2000). In microsporogenesis, proper tapetum development and differentiation is essential for pollen fertility, while in meiosis and megagametogenesis, proper tapetal PCD is vital for pollen fertility. Several genes are identified for tapetal PCD and pollen development, including persistent tapetal cell 2 (*PTC*2), helix–loop–helix (*bHLH*) transcription factors, tapetum degeneration retardation (*TDR*, *bHLH5*), undeveloped tapetum 1 (*UDT1*, *bHLH164*), eternal tapetum 1/delayed tapetum degeneration (*EAT1*/*DTD*, *bHLH141*), TDR-interacting protein 2 (*TIP2*, *bHLH142*), MYB family transcription factor *GAMYB*, PHD-finger protein persistent tapetal cell 1 (*PTC1*), TGA transcription factor *OsTGA10*, glycerol-3-phosphate acyltransferase 3 (*OsGPAT3*), and degenerated panicle and partial sterility 1 (*DPS1*) (Sun et al., 2018; Uzair et al., 2020; Zafar et al., 2020). Specifically, *TDR*, *TIP2*, *EAT1*, *PTC2*, and *OSGPAT3-2* exhibit delayed PCD, leading to an abnormal pollen wall and eventually sterile phenotype (Sun et al., 2018; Yang et al., 2019a; Uzair et al., 2020)*.*

This study reported novel RNA recognition motifs containing protein, named *DCET1* (defective in callose, exine and tapetum1). *Dcet1* mutant showed small whitish anthers with irregular outer and inner surfaces having sterile pollens due to defective callose biosynthesis and degeneration, improper pollen exine patterning, and delayed tapetal PCD. Additionally, *DCET1* showed higher expression in anthers, specifically at stages 6–8 of anther development, confirming its involvement in callose and pollen wall development. Our results provide the first evidence of the RRM/RBD family protein *DCET1* in rice male sterility through defective callose, exine wall, and tapetal cell PCD, which justifies its role in hybrid rice breeding. Also, being the closest homolog of *Arabidopsis* CID-like proteins of mainly unknown functions, *DCET1* receives key importance for predicting and characterizing RBDs in general and CIDs in specific in all eukaryotes.

## Materials and Methods

### Plant Materials

The *dcet1* mutant was identified from an ethyl methanesulfonate (EMS)–induced mutant library of an indica rice cultivar Zhonghui 8015. The complete male-sterile mutant *dcet1* was crossed with the wild-type (WT) and 02428 (sp. japonica), respectively. F_1_ heterozygous plants were self-pollinated to obtain BC_1_F_2_ and F_2_ populations for genetic analysis and mapping. All the plants were grown in paddy fields of the China National Rice Research Institute Hangzhou, Zhejiang Province, and Lingshui, Hainan Province, China.

### Phenotypic Observation of WT and *dcet1* Mutant

The phenotypes of the whole WT and *dcet1* mutant lines were photographed using Nikon HB-40, Japan. The reproductive organs were captured with a Carl Zeiss Stereo Lumar V12 stereo fluorescence stereomicroscope (Markku Saari, Jena, Germany). To assess the pollen viability, mature anthers were collected from the WT and *dcet1* mutant and stained with 1.2% I_2_-KI solution. The samples were observed and imaged using a Leica DM2500 microscope.

### Cytological Observation

For semi-thin sections, spikelets of both WT and *dcet1* at different developmental stages were collected and fixed in a formalin–acetic acid–alcohol (FAA) solution (1:1:18) followed by dehydration using a graded series of 50–100% ethanol as described previously (Sun et al., 2018). After dehydration, the samples were embedded using Technovit glycol methacrylate 7100 resin (Heraeus, Kulzer, Germany), and polymerized at 50°C. Semi-thin sections of 2 µm were cut using an RM2265 rotary microtome (Leica, Germany) and stained with 0.1% (w/v) toluidine blue. The stained sections were observed and imaged using the Leica DM2000 microscope.

For transmission electron microscopy (TEM) analysis, the spikelets of WT and *dcet1* were pre-fixed in 2.5% glutaraldehyde in phosphate buffer (0.1M, pH7) overnight at 4°C. The samples were then rinsed thrice using phosphate-buffered saline (PBS; 0.1M, pH = 7.2) for 15 min at each step. The specimens were then post-fixed for 1.5 h with 1% OsO_4_ in phosphate buffer and rinsed three more times as before, followed by dehydration using ethanol series (30–100%). After dehydration, the samples were infiltrated in a 1:1 mixture of absolute acetone and the final Spurr’s resin for 1 h at room temperature, followed by a 1:3 mixture for 3 h, and the final Spurr’s resin overnight. Following infiltration, the specimens were placed in Eppendorf contained Spurr’s resin and heated at 70°C for more than 9 h. Ultra-thin sections were cut in a LEICA EM UC7 ultratome (Germany) and double-stained with 2% uranyl acetate and 2.6% alkaline lead citrate aqueous solution for 5–10 min, respectively. The stained sections were pictured with a Hitachi Model H-7650 transmission electron microscope (Japan) at the Center of Electron Microscopy, Zhejiang University (Hangzhou, China).

Scanning electron microscopy (SEM) analysis was performed by fixing the mature anthers of WT and mutant with 2.5% glutaraldehyde in 0.1 M sodium phosphate buffer at 4°C. The samples were then dehydrated with the same graded ethanol series (30–100%) as performed in TEM, and exchanged three times with isoamyl acetate. The fixed samples were then critical point-dried, gold-coated, and mounted. The samples were observed and photographed using a scanning electron microscope (Hitachi TM-1000) at the Center of Electron Microscopy, Zhejiang University (Hangzhou, China) with an accelerating voltage of 10 or 15 kV.

### TUNEL Analysis

The terminal deoxynucleotidyl transferase-mediated dUTP nick-end labeling (TUNEL) assay was carried out to scrutinize the tapetal cell PCD. Paraffin sections of the WT and *dcet1* anthers at different developmental stages were prepared and processed as described previously (Chang et al., 2014). The suitable paraffin sections were dewaxed in xylene and rehydrated in a graded ethanol series. The TUNEL assay was performed using the TUNEL Bright Green Apoptosis Detection Kit A-112 (Vazyme Biotech Co., Ltd.) according to the supplier’s instructions with little modifications. The TUNEL (green fluorescence, at a wavelength of 488 nm) and DAPI signals (blue fluorescence, at a wavelength of 405 nm) were observed and imaged using a fluorescence confocal scanner microscope (ZEISS LSM 700, Jena, Germany).

### Meiotic Chromosome Preparation

For meiotic observation, 4′,6-diamidino-phenylindole (DAPI) staining was carried out by sampling the young meiosis stage spikelets of both WT and *OSRRMS1.* The samples were fixed in Carnoy’s solution (ethanol: glacial acetic, 3:1) and processed according to the previous prescription (Wu et al., 2015).

### Aniline Blue Staining for Callose

For callose staining of WT and *dcet1*, anthers at different developmental stages were collected and stained through 0.1% aniline blue for 15 min at 4°C. The samples were prepared and processed as described previously (Lu et al., 2014). The presence of callose (blue-yellow fluorescence) was visualized under an excitation wavelength of 420 nm and emission wavelength of 530 nm using a fluorescence confocal microscope. The same setting was used across all samples.

### *DCET1*-Promoter-GUS Assay

GUS staining was observed by sampling fresh tissues and anthers at different developmental stages from transgenic plants expressing the *DCET1*-promoter-GUS construct and stained as described previously (Zou et al., 2017a). The samples were washed in ethanol and pictured by using a light microscope.

### Gene Mapping of *DCET1*

For genetic mapping, the *dcet1* mutant (sp. indica) was crossed with 02428 (sp. japonica) to get F_1,_ which was further grown to derive the F_2_ population. A total of 1,050 F_2_ male-sterile plants were used for genetic mapping. For fine mapping of *DCET1*, SSR (simple sequence repeat) and InDel (insertion–deletion) markers were developed based on polymorphism between parents. After fine mapping and cloning of the targeted *DCET1*, 30 homozygous sterile plant DNA of the *dcet1* F_2_ population were used for MutMap to verify the mapping results further. The MutMap technique was employed according to the previous prescription (Abe et al., 2012).

### Sequence and Phylogenetic Analysis

The amino acid full-length sequence of *DCET1* and selected *Arabidopsis* CIDs and other 17 gene sequences were retrieved with BLASTP (http://www.ncbi.nlm.nih.gov/), and conserved domains were searched in CD-search (https://blast.ncbi.nlm.nih.gov/Blast.cgi). Because *DCET1* belongs to a CID-like protein, first *Arabidopsis* CID group-D genes and second 17 other amino acid sequences were selected for multiple sequence alignments and evolutionary analysis. Sequence alignments were carried out using Clustal Omega (https://www.ebi.ac.uk/Tools/), and the Neighbor-Joining method (Saitou and Nei, 1987). Resultant alignments were used to construct the maximum likelihood trees by using Mega X with 1000 bootstrap replicates (Kumar et al., 2018).

### Vector Construction

For the complementary vector, an entire 10,249 bp region was amplified from the WT (ZH8015) genomic DNA; consisting of the entire ORF of LOC_Os08g02330, 2,943 bp upstream sequence and 1,043 bp downstream sequence by using the primers *DCET1*-CF and *DCET1*-CR (Supplementary Table S1). The fragment was then cloned into binary vector pCAMBIA1300 using the In-Fusion HD Cloning Kit (Takara Bio USA Inc., Mountain View, CA, United States).

To gain further insights into the role of LOC_Os08g02330 in rice male sterility, we designed a 23 bp target in the 2nd exon within this gene (Supplementary Table S1). Vector construction was carried out by using the CRISPR/Cas9 genome editing system in ZH8015 indica background to investigate its function as previously described (Zou et al., 2017b), with some modifications. We also observed the CRISPR/Cas9 transgenic plants for the target site mutation through direct or cloned sequencing of the PCR products by using site-specific primers (Supplementary Table S1).

For the GUS vector, a 3,074 bp promoter of *DCET1* was amplified from the WT genomic DNA, using the primers *DCET1*-GF and *DCET1*-GR (Supplementary Table S1). The fragment was cloned into the binary vector pCAMBIA1305 with BamH1 and Nco1 restriction sites using an In-Fusion HD Cloning Kit (Takara Bio USA Inc., Mountain View, CA, United States).

### RNA Isolation, cDNA Synthesis, and Real-Time Quantitative Reverse Transcription PCR

Total RNA was isolated from different rice tissues using the RNAprep Pure Plant Kit (Tiangen, Beijing, China), according to the manufacturer’s instructions. RNA concentration was adjusted and reverse-transcribed into cDNA using the ReverTra Ace^®^ qPRT-PCR Master Mix with gDNA Remover (Toyobo, Japan). For the reaction and qRT-PCR, LightCycler 480 (Roche, Germany) using LightCycler^®^ 480 SYBR^®^ Green I Master Mix (Roche, United States) was used according to the standard manufacturer’s recommended program and guidelines. In all samples, ubiquitin is used as an internal control, and each reaction was repeated three times (Zhang et al., 2017).

### Analysis of Anther Wax and Cutin Monomers

For anther cuticular lipids, WT and *dcet1* anthers at stage 12 were collected and immediately frozen in liquid nitrogen. The wax and cutin contents were determined and analyzed as previously described (Shi et al., 2011; Zhu et al., 2013). Statistical analysis was carried out by using Student’s *t*-test.

## Results

### Characterization of the *DCET1* Mutant

**FIGURE 1:**
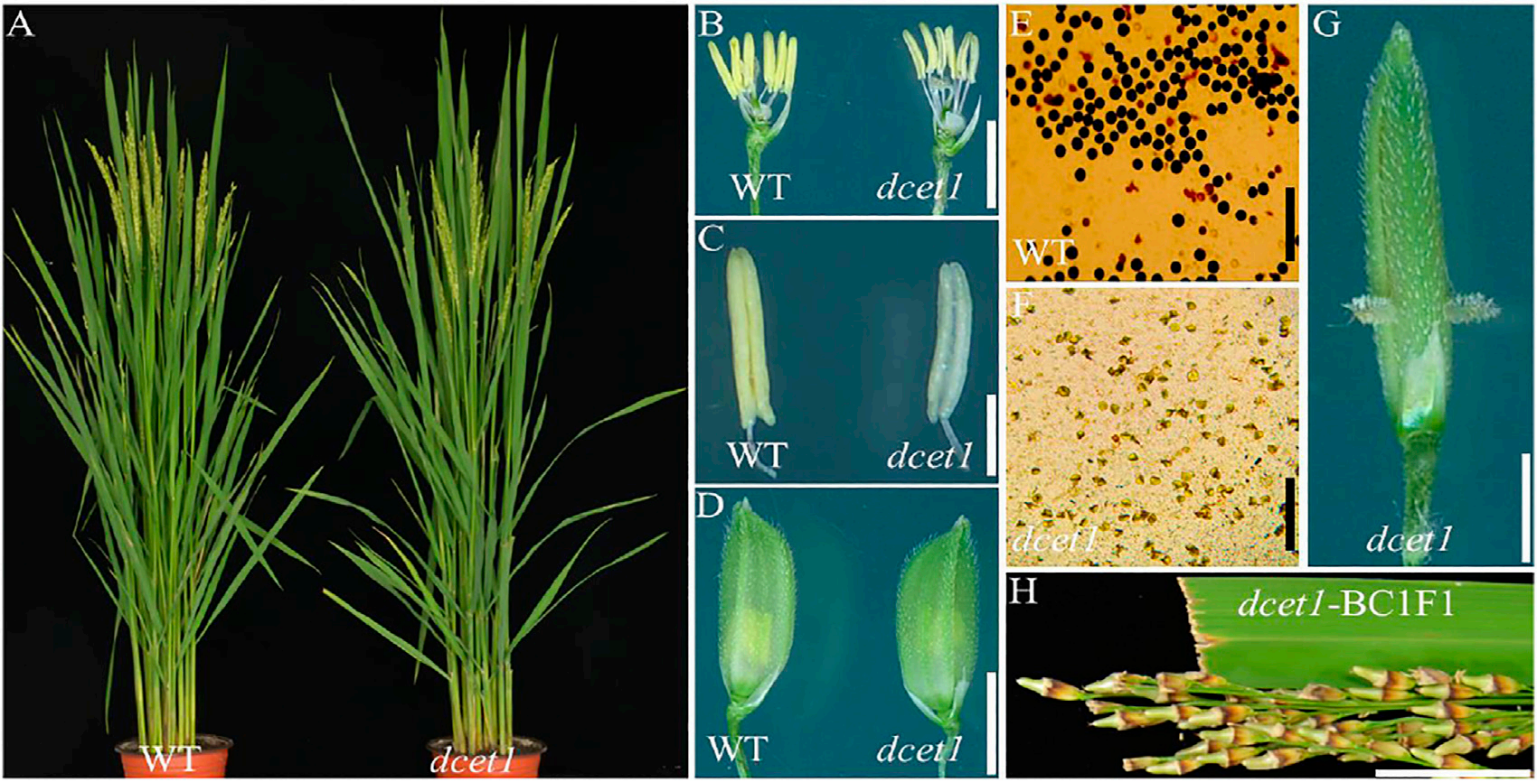
Phenotypic comparison of wild-type (WT) and mutant (*dcet1*). Plants after heading **(A)**; Spikelets after the removal of palea and lemma **(B)**; Anthers at stage 13 **(C)**; Spikelets at the heading stage **(D)**; Pollen grains after staining with 1.2% I2-KI staining solution **(E,F)**; Spikelets of *dcet1* with stigma extrusion **(G)**; Panicle of *dcet1*-BC1F1 **(H)**. Scale bars = 20 mm in **(A)**, 1 mm in **(B,C)**, 2 mm in **(D,G)**, 100 µm in **(E,F)**, and 1 cm in **(H)**.

Mutant *dcet1* was screened from EMS treatment of an *Indica* background cultivar Zhonghui8015 (ZH8015). All vegetative and agronomic traits of the *dcet1* mutant, including plant height and general floral morphology, were the same as that of wild-type ZH8015 (WT), except the anthers (Figures 1A–C). The *dcet1* anthers were weaker, smaller and whitish-yellow as than the WT having dark yellow and normal-sized anthers (Figures 1B–D). After staining with I_2_-KI, the mutant *dcet1* was found to be completely sterile with whitish-yellow and hill-shaped pollen grains, but fertile WT pollen grains were dark-black and round-shaped (Figures 1E,F). Additionally, the extrusion of stigma was also observed in the *dcet1* mutant plants (Figure 1G). The BC_1_F_1_ plants of the mutant cross with WT were completely fertile (Figure 1H), and F_2_ plants showed a fertile/sterile segregating ratio of 3:1 (168 fertile and 58 sterile plants), confirming the recessive mutant nature of *dcet1*. These results also ensure that the female part is fertile in the *dcet1* mutant.

### Male Reproductive Defects of the *dcet1* Mutant

**FIGURE 2:**
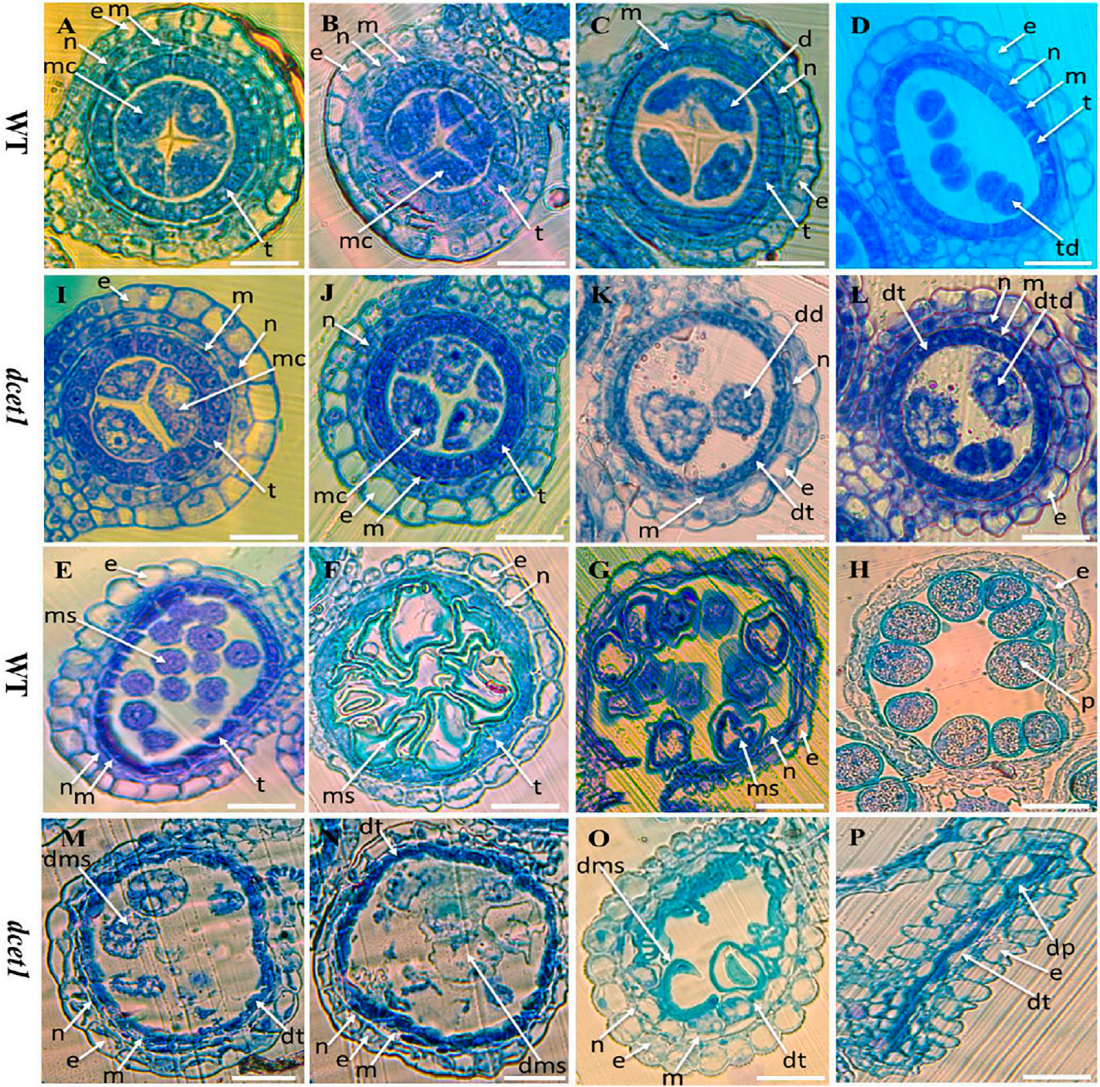
Semi-section analysis of wild-type (WT) and mutant (*dcet1*). Anther sections of WT **(A–H)** and *dcet1* **(I–P)** from stages 6–10 and 12 and 13 of anther development, respectively. e, *epidermis*; n, endothecium; m, middle layer; t, tapetum; mc, meiocyte; dt, defective tapetum; d, dyad; dd, defective dyad; td, tetrad; dtd, defective tetrad; ms, microspore; dms, defective microspore; p, pollen; dp, defective pollen. Scale bars = 15 µm.

To explore the reproductive system defects of the *dcet1* mutant, we investigated the cytological mechanism of WT and *dcet1*. The anther development was divided into 14 stages based on the length as prescribed previously (Zhang et al., 2011). The semi-thin transverse sections of the different developmental stages from the anthers of *dcet1* were compared with those of WT (Figures 2A,P). No obvious defects were traced till the formation of microspore mother cells (MMCs) from the secondary sporogenous cells (Figures 2A,I). Typically at this stage of anther development, the anther locule is encircled by the four-layered anther wall (Zhang et al., 2011). Prominent abnormalities were observed with the initiation of meiotic division stage 7, when the WT meiocytes were darkly stained, well-developed, and spherical-shaped, but *dcet1* mutant meiocytes were less stained, hollow, and variable-shaped, as described in Figures 2B,J. However, at the corresponding stage, the *dcet1* anthers produced morphologically normal four somatic layers of epidermis, endothecium, middle layer, and tapetum, like those of WT (Figures 2B,J). At stage 8a of anther development, the meiocytes undergo meiosis, and the anther initiates the tapetal cell programmed cell death (PCD). Meanwhile, the WT microspores were divided into dyads separated by the cell plate, and the tapetum became more vacuolated and condensed (Figure 2C). In contrast to the WT, in the *dcet1* mutant, the formation of dyads was completely irregular, the microspores changed into a conical and bursted shape, and the tapetum was less vacuolated and had not expanded properly (Figures 2C,K). At stage 8b, the WT meiocytes developed into dark-stained tetrads and the tapetum was further vacuolated, but the *dcet1* microspore burst out and became less stained with variable vacuoles inside (Figures 2D,L). In addition, the middle layer was nearly invisible in WT anthers, while in *dcet1*, it was still clearly apparent with negligible signs of degradation (Figures 2D,L). During stage 9, the WT microspores were released from the tetrad and evenly spread inside the anther locule (Figure 2E). However, the *dcet1* microspores were degenerated, shrinked, or over-broadened and looked unable to produce viable pollen grains (Figure 2M). Here the *dcet1* tapetum was lightly stained and not properly vacuolated as compared to that of WT, indicating abnormal PCD (Figures 2E,M). Furthermore, at stage 10, the WT-vacuolated and teeth-like microspores were almost attached with the tapetum leaving a circle-like space at the middle of locule; but the *dcet1* microspores were asymmetrical, non-vacuolated, and grass-like residues spread inside the anther locule (Figures 2F,N). Correspondingly, the WT tapetum was degenerated and hill-like, whereas the *dcet1* tapetum was still properly intact and exhibited the delayed nature of *dcet1* tapetal cell PCD (Figures 2F,N). Eventually, at mature stages of anther development, when the WT released the normal and viable pollen grains, the *dcet1* mutant produced shriveled and debrised pollen grains inside the anther locule with complete sterility (Figures 2G,H,O,P).

**FIGURE 3:**
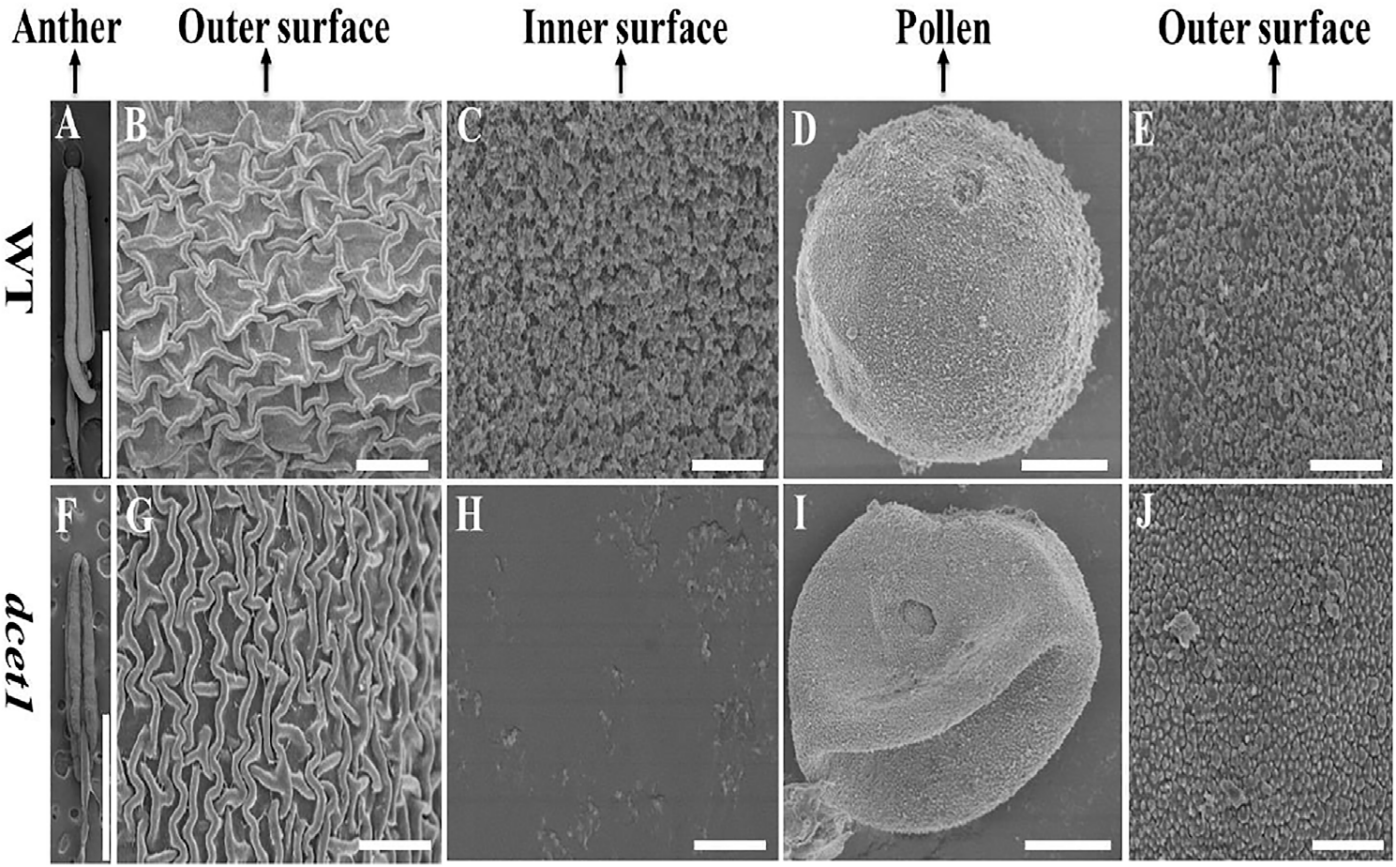
Scanning electron microscopy (SEM) analysis of the surfaces of anthers and pollen grains in the wild-type (WT) and mutant (*dcet1*) **(A–E)** Represents WT and **(F–J)** represents *dcet1*. Scale bars = 100 µm in **(A,F)**, 2 µm in **(B,C,G,H,E,J)**, and 1 µm in **(D,I)**.

To further confirm the male reproductive and morphological defects of the *dcet1* mutant, we performed the SEM analysis of mature anthers (stage 13) for both WT and *dcet1*. In conformity with the abovementioned phenotypic results, the anthers were smaller in *dcet1*, having collapsed nanoridges on the anther outer surface than WT, typically having prominent and organized nanoridges on the anther cuticle outer surface (Figures 3A,B,F,G). More obvious differences were found on the inner surface of the anther between the WT and *dcet1* mutant. The inner surface of the WT anther was covered by evenly distributed Ubisch bodies, while the *dcet1* anther inner surface was almost missing in the Ubisch bodies (Figures 3C,H). The WT plant pollen grain was round globe-shaped with an organized lobed exine surface; in contrast to WT, the pollen of *dcet1* was shrunken, with irregular and softened outer exine (Figures 3D,E,I,J). These results indicated the abnormalities of anther and pollen and their surfaces in the *dcet1* mutant.

**FIGURE 4:**
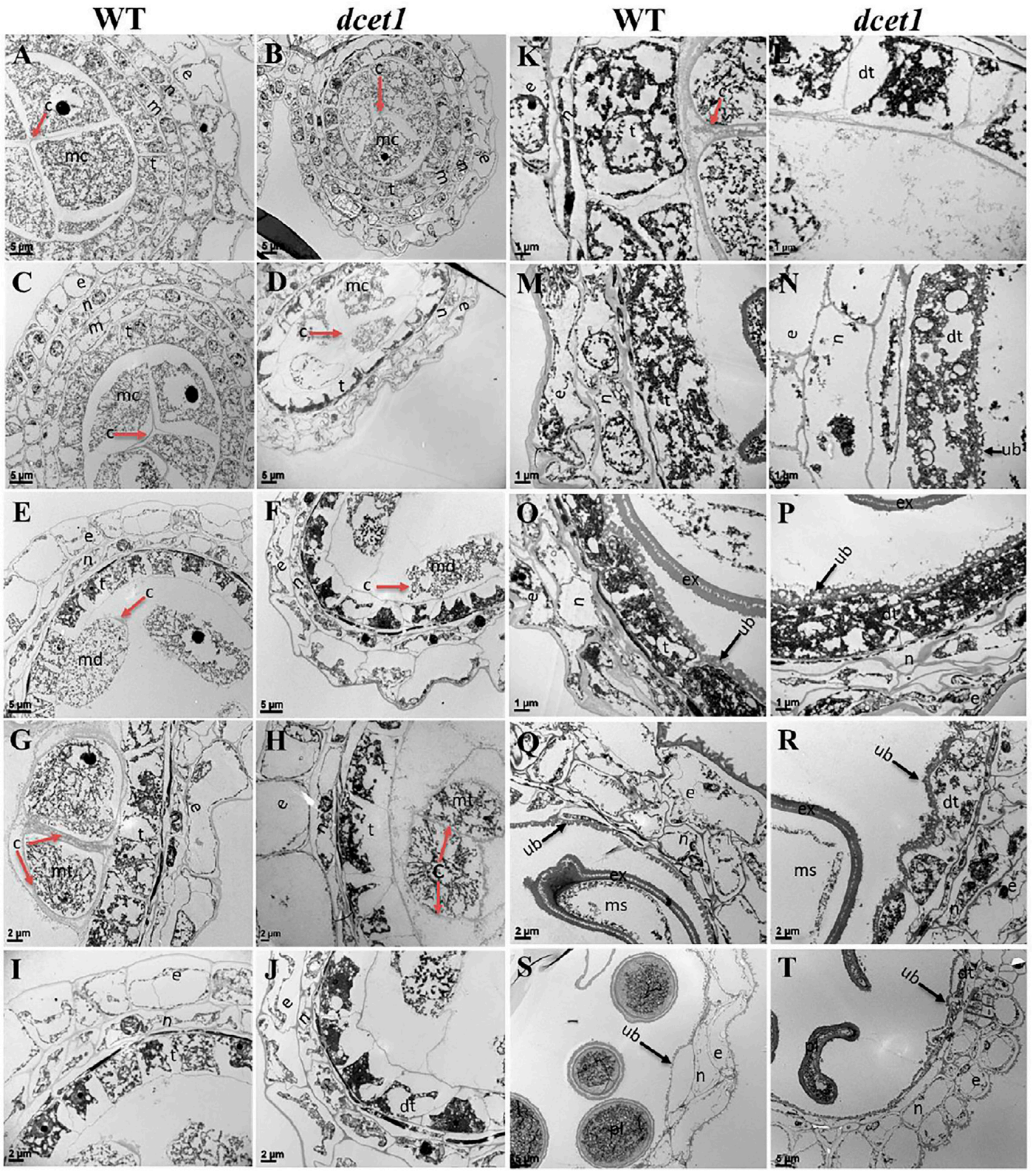
TEM analysis of the anther callose wall and tapetum in the wild-type (WT) and mutant (*dcet1*). Comparison of WT and *dcet1* for the callose wall **(A–H)**; Comparison of WT and *dcet1* for the tapetum **(I–T)**. Red and black arrows are used for describing the callose layer and tapetum Ubisch bodies, respectively. c, callose; mc, mother cell; md, dyad; mt, tetrad; ms, microspore, dms, defective microspore; pl, pollen; dpl, defective pollen; e, *epidermis*; n, endothecium; m, middle layer; t, tapetum; dt, defective tapetum; ub, Ubisch bodies. Scale bars = 5 µm **(A–F,S,T)**, 2 µm **(G–J,Q,R)**, and 1 µm **(K–P)**.

To further elaborate the differences between WT and *dcet1*, we examined the different stages of anther development of both WT and *dcet1* mutant through transmission electron microscopy (TEM). No obvious changes were observed until the sporogenous cell divides into secondary sporogenous cells and until the formation of anther somatic layers (endothecium, middle layer, and and tapetum) between the WT and *dcet1*. However, a stronger callose layer was seen in WT, to that of mutant *dcet1* during this period (Figures 4A,B). But the most distinguished abnormalities were observed at stage 7 and onward, when the microspores start differentiating from each other through callose wall formation. At this stage, an off-white–colored callose layer could be observed in the WT anther locule differentiating the microspores, while in the *dcet1* mutant; this callose layer was not clearly visible, showing the male reproductive defective nature of *dcet1* before proper initiation of meiosis (Figures 4C,D). In addition to callose layer defects between WT and mutant *dcet1* at the corresponding stage, the WT meiocytes were electron-densely stained and compact in shape; in contrast, the *dcet1* meiocytes were thinly stained having variable vacuoles and hollow cavities inside (Figures 4C,D). In semi-thin transverse section studies, the same defects were observed at this stage in *dcet1* anthers. At early stage 8a, the callose wall surrounded the meiocytes in WT, while in *dcet1*, the callose wall was missing or degenerated, favoring the abnormalities (Figures 4E,F). Similarly the surface was irregularly stained in *dcet1* as compared to WT (Figures 4E,F). At later stage 8b, the WT tetrads were generated and separated by the thick callose layer, while in *dcet1* no organized callose wall was observed; only an irregularly curled thin line could be seen surrounding the defective dyads (Figures 4G,H). TEM analysis also confirmed the delayed tapetum degeneration and programmed cell death (PCD) in the *dcet1* mutant. The *dcet1* anthers did not differ from those of WT for tapetum development and other somatic layers till the dyad formation stage 8a (Figures 4I,J). At the tetrad stage 8b, the WT tapetum started to degrade through proper PCD and underwent extended vacuolation (Figure 4K). In contrast to WT, the *dcet1* mutant tapetum was weakly stained, covered with a thick cell wall, and produced decreased vacuolation (Figures 4K,L). At stage 9, the WT tapetum continued to degrade and looked thinner, while the *dcet1* mutant tapetum was devacuolated and formed a bubble-like structures spread over the tapetum surface (Figures 4M,N). At stage 10, both the WT and *dcet1* mutant formed normal Ubisch bodies; however, the *dcet1* tapetum was wider and darkly stained with negligible vacuolation than that of WT (Figures 4O,P). At stage 11, when the WT tapetum was completely dissolved, the *dcet1* tapetum could be easily observed, and even at the mature stage, the thinner mutant tapetum could be seen (Figures 4Q–T).

**FIGURE 5:**
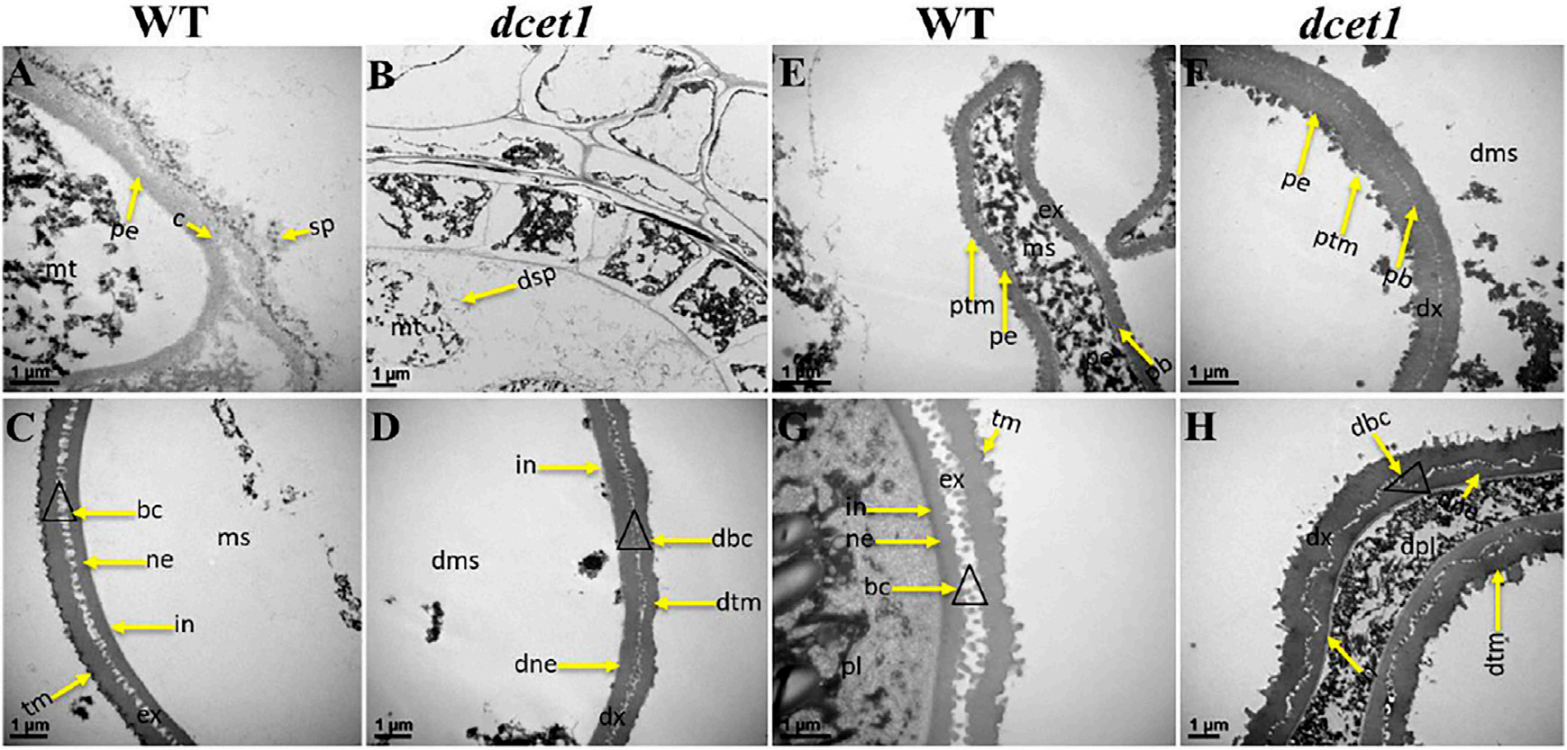
TEM analysis of pollen wall development in the wild-type (WT) and mutant (*dcet1*). Comparison of WT **(A,C,E,G)** and *dcet1* **(B,D,F,H)**. mt, tetrad; ms, microspore, dms, defective microspore; pl, pollen; dpl, defective pollen; pe, primexine; sp, sporopollenin; dsp, defective sporopollenin; ex, exine; dx, defective exine; ne, nexine; dne, defective nexine; pb, probocula; bc, bacula; dbc, defective bacula; tm, tectum; ptm, protectum; dtm, defective tectum; tr, tryphine; dtr, defective tryphine; in, intine. Scale bars = 1 µm.

Furthermore, we had also observed the pollen exine patterning in both WT and *dcet1* mutant through TEM analysis. The results showed obvious changes between WT and *dcet1* mutant at the tetrad stage (8b). At this stage, we had observed the deposition of sporopollenin precursors on the primexine surface in the shape of black dots, while no such deposition was seen in the *dcet1* mutant (Figures 5A,B). At stage 9 in WT, a well-organized protectum was observed in the form of darkly stained protrusions on the microspore outer surface; in contrast, the *dcet1* microspore surface was having a detached and irregularly distributed protectum (Figures 5E,F). At stage 10, the exine of the WT microspore wall was thickly stained, having a regular two-layered surface with typical exine patterning, while the exine of the *dcet1* microspore was thinly stained with deformed bacula, defective tectum, and irregular nexine (Figures 5C,D). Furthermore, at the mature stage (stage 13), in contrast to the spherical pollen grains of WT which were having normal exine with well-formed bacula, protruded tectum, and normal nexine, the exine of *dcet1* pollen was malformed with disintegrated bacula, defective tectum, and misshaped nexine (Figures 5G,H). TEM results demonstrated the abnormal and abortive nature of *dcet1* pollen grains through defective callose regulation, improper exine patterning, and delayed tapetal cell PCD.

### Callose Wall During Microsporogenesis Was Defective in *dcet1*

**FIGURE 6:**
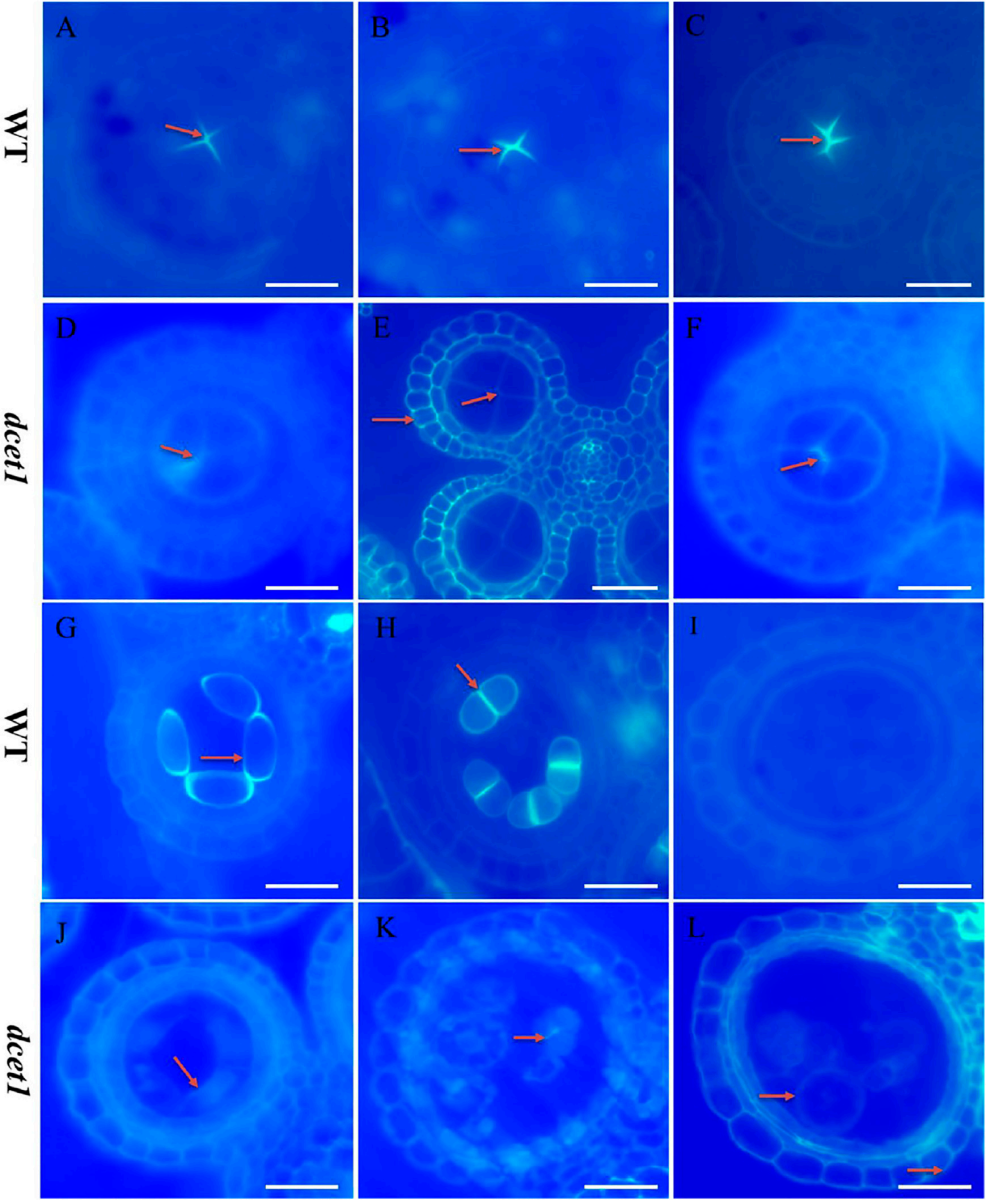
Anther callose layer analysis of wild-type (WT), and mutant (*dcet1*). Aniline blue staining of WT **(A–C,G–I)** and *dcet1* **(D–F, J–L)** for anther development stages 5, 6, 7, 8a, 8b, and 9, respectively, Red arrows highlight the callose layer. Scale bars = 20 µm.

During TEM analysis, the abnormalities in callose metabolism were observed in the *dcet1* mutant. Thus we performed the aniline blue staining of both WT and *dcet1* anther sections during various stages of anther development. Both WT and *dcet1* mutant meiocytes were nearly normal for pre-callose wall synthesis; however, the weakly signals were observed in *dcet1* anthers compared to WT at stage 5 of anther development (Figures 6A,D). This specified that callose synthesis was prominently affected in the *dcet1* mutant at the start (Figures 6A,D). Moreover, no other obvious changes were observed in the *dcet1* mutant than WT until stage 6 of anther development, except the weakest callose layer signals and its irregular distribution over the *dcet1* mutant microsporocyte surface within the anther locule (Figures 6B,E). At anther stage 7, WT microspores were separated by a thickened and well-formed callose wall (Figure 6C). Meanwhile in the *dcet1* anther, some amount of callose was accumulated on the surface of the four somatic layers of the anther wall, and the meiocytes had such weaker signals for callose wall accumulation that only a few meiocytes could be hardly recognized (Figure 6F). At stage 8a, when the WT meiocytes developed dyads, they were separately well-surrounded by the callose wall, but *dcet1* mutant anthers almost had no callose wall, and a thinner callose whitish layer was observed spread over the whole surface of the meiocytes and anther wall (Figures 6G,J). At stage 8b of anther development, WT meiocytes were divided into tetrads through meiosis, properly encircled from the outside as well as within the tetrads separately, but the *dcet1* mutant meiocytes underwent no such division due to impaired and degenerated callose wall synthesis as presented in Figures 6H,K. After meiosis at stage 9, when the WT microspores were properly released due to timely degeneration of the callose wall through callases and initiation of the pollen wall, the *dcet1* microspores still had a thinner and defective callose wall, and hence abnormal pollen grains were produced (Figures 6I,L). These results suggested that the synthesis and degradation of the callose wall was a continuous process in the WT. However, in *dcet1*, a thinner or defective callose wall staining was observed, and even after the tetrad stage, some amount of callose was seen spread over the anther locule, resulting in abnormal pollen grains and causing fusion among sibling microspores. These findings indicated that callose biosynthesis and dissolution were defective and abnormal in the *dcet1* mutant.

### Meiosis in the *dcet1* Mutant

To check whether meiosis in the *dcet1* mutant was defective or not with the defects in callose accumulation, we carried out aniline blue and DAPI stainings for callose and meiosis at different anther development stages. In the WT and mutant *dcet1*, no changes were observed in meiosis from the start till the production of tetrads. At diakinesis, both WT and *dcet1* condensed normally and produced paired chromosomes into 12 bivalents and were aligned on the equatorial plate during metaphase I (Supplementary Figures S1A–C,E–G). As in WT, anaphase I was also perceived to be standard for *dcet1* by migrating the homologous chromosomes into opposite poles, and finally dyads were formed at the completion of meiosis I (Supplementary Figures S1D,H,I,M). During meiosis II, parallel to the WT, in the *dcet1* chromosome, the sister chromatids were separated normally and produced normal tetrads (Supplementary Figures S1L,P). Although both WT and *dcet1* microsporocytes appeared normal for meiosis, the callose accumulation was not normal, and a thinner and scattered callose layer was observed in the *dcet1* mutant than WT (Figures 6A–L; Supplementary Figures S1A–P). At meiosis stage 8a and b, the microspores could be observed separately and showed a normal nuclear division of the mutant male gametophyte, although the surrounding callose wall is invisible in the *dcet1* mutant (Figures 6G,H,J,K; Supplementary Figures S1A–P). However, most of the *dcet1* young pollens were observed to be swollen and bursted due to the abnormal callose wall (Figures 6I,L), irrespective of normal meiosis, confirming the key role of callose for pollen maturation and viability.

### Delayed DNA Fragmentation in *dcet1* Tapetal Cells

**FIGURE 7:**
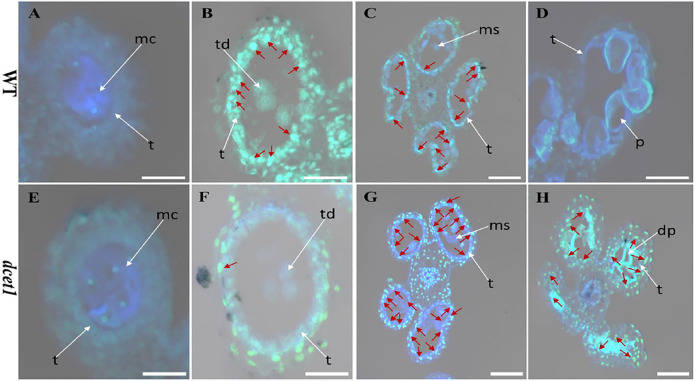
TUNEL analysis of wild-type (WT) and mutant (*dcet1*). Anthers of WT **(A–D)**, and *dcet1* **(E–H)**, for pre-meiosis, meiosis, post-meiosis, and mature pollen stages of anther development, respectively. Red arrow highlights the strength of TUNEL signals. t, tapetum; mc, meiocyte; td, tetrad; ms, microspore; p, pollen; dp, defective pollen. Scale bars = 20 µm.

The *dcet1* mutation showed the defective degradation of somatic walls during cytological analysis. Thus, we assumed that the abortive anthers of the *dcet1* mutant would have abnormal PCD. Therefore, we performed the terminal deoxynucleotidyl transferase-mediated dUTP nick-end labeling (TUNEL) assay in WT and *dcet1* mutant at different stages of anther development as previously described (Uzair et al., 2020). Before stage 8 of anther development, both the WT and *dcet1* mutant had no DNA fragmentation signals (Figures 7A,E). DNA fragmentation signals were first observed at meiotic stage 8 in the WT tapetal cells, but no such signals were visualized during this stage in the *dcet1* mutant (Figures 7B,F). At stage 9, when the microspores were released, signals for DNA fragmentation appeared in the *dcet1* mutant and reached its peak till the later stages of anther development (Figures 7G,H). On the other hand, the DNA fragmentation in the WT occurred in a typical manner and ended at stage 11 (Figures 7C,D). These results further confirmed the delayed PCD of the tapetal cells in the *dcet1* mutant.

### Analysis of Anther Cuticular Lipids

**FIGURE 8:**
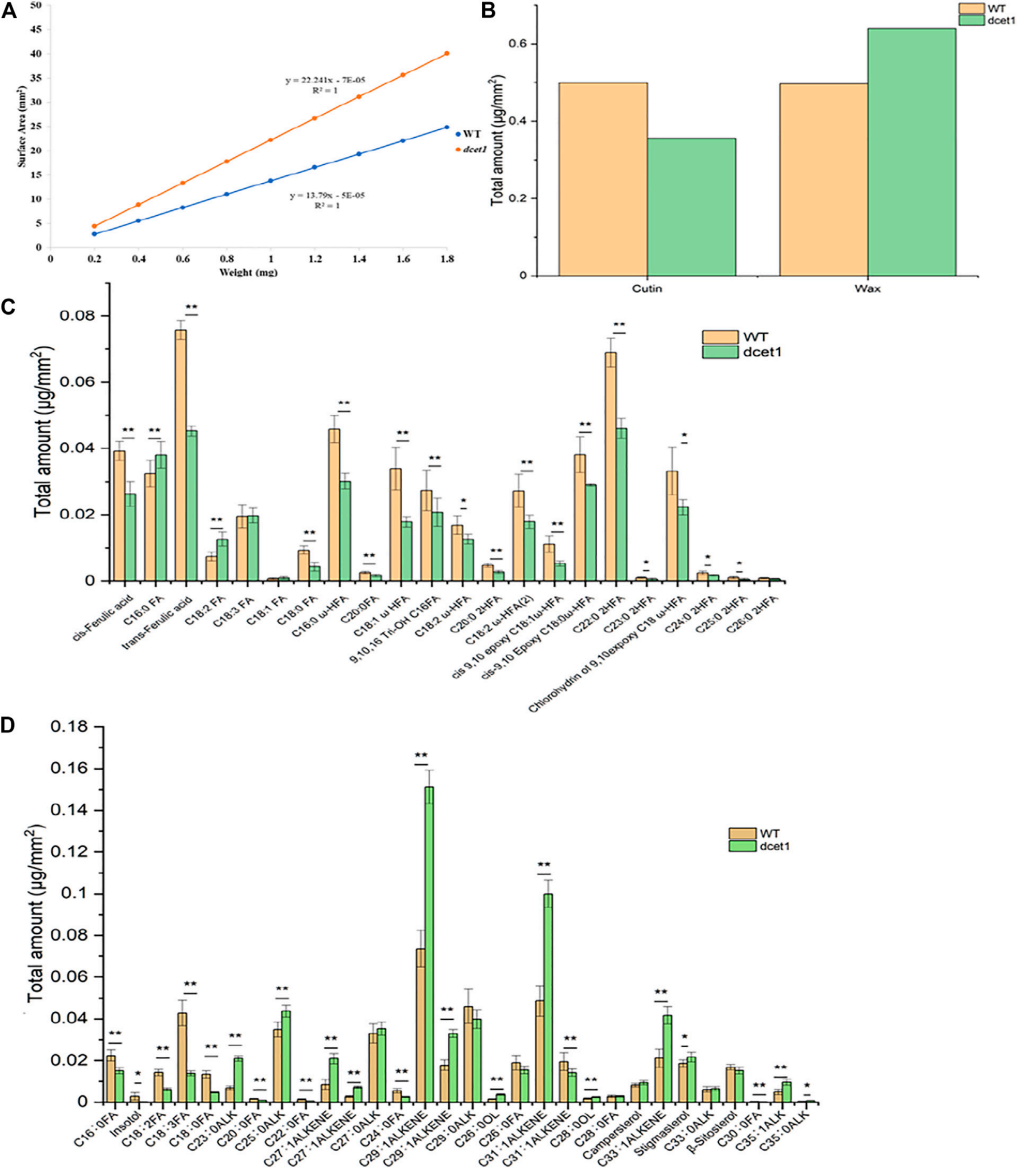
Analysis of anther wax and cutin in the wild-type (WT) and mutant (*dcet1*). Weight/surface area ratio of the WT and *dcet1* anthers **(A)**. Total wax and cutin amounts per unit surface area in WT and *dcet1* anthers **(B)**. Cutin monomer amount per unit surface area in WT and *dcet1* anthers **(C)**. Wax constituent amount per unit surface area in WT and *dcet1* anthers **(D)**. *R* ^2^ represents regression values. Error bars indicate ± SD (*n* = 5). *p-value < 0.05; **p-value< 0.01 by Student’s *t*-test.

TEM analysis had revealed that the *dcet1* pollen wall was comparatively thick at the later stages of anther development (Figures 5G,H). Simultaneously, SEM results indicated a subtle change in the anther outer surface between *dcet1* and WT (Figures 3B,G), encouraging the comprehensive analysis of anther cuticular lipids. The cuticular wax constituents and cutin monomers in both WT and *dcet1* anthers at stage 12 were measured by gas chromatography-mass spectrometry (GC-MS) and gas chromatography-flame ionization detection (GC-FID). The amounts per unit area were determined by plotting the calculated anther surface area against the dry weight of each corresponding sample (Figure 8A). The results showed that the total amount of cutin monomers on the *dcet1* anther was 0.356 μg mm^−2^ compared to 0.499 μg mm^−2^ on the WT anther surface, which corresponds to a highly significant reduction in total cutin (Figures 7B,C). The cis and trans-ferulic acids, the hydroxy fatty acid C23:0 2HFA, the unhydroxylated fatty acid C18:2 FA, and ω-hydroxy fatty acids, such as C16:0 ω-HFA, C18:1 ω HFA, C18:2 ω-HFA (2), cis-9,10 epoxy C18:0 ω-HFA, and chlorohydrin of 9,10expoxy C18 ω-HFA, were dominant cutin monomers, and were found at significantly lower levels in *dcet1* anthers (Figure 8C). Most of the unhydroxylated fatty acids, such as C18:1 FA, C18:0 FA, C20:0FA, C16:0 FA, and C18:3 FA, and hydroxy fatty acids, such as C20:0 2HFA, C23:0 2HFA, C24:0 2HFA, C25:0 2HFA, and C26:0 2HFA, were found in very less or in non-significant amount (Figure 8C). The total amount of wax in the *dcet1* anther was 0.639 μg mm^−2^, whereas the wild-type anther wax was 0.497 μg mm^−2^, which corresponds to a highly significant increase in total wax content of the anther (*p-value* < 0.01) (Figure 8D). This increase of cuticular wax content in *dcet1* anthers was mainly attributed to the significant rise in alkene content, such as C27:1ALKENE, C29:1ALKENE, C31:1ALKENE, C33:1ALKENE, and a few alkanes such as C23:0ALK, C25:0ALK, and C35:1ALK (Figure 8D). The data also showed the decrease in almost all recorded fatty acids for *dcet1* anthers, while the other wax constituents are mainly non-significant (*p-value* < 0.01) (Figure 8D). The wax and cutin analysis of *dcet1* suggested that *DCET1* is involved in regulating the biosynthesis of lipidic compounds to form anther cuticle and pollen exine in rice, and the denser pollen exine at later stages might be due to the increase of wax constituents.

### Cloning of *DCET1*

**FIGURE 9:**
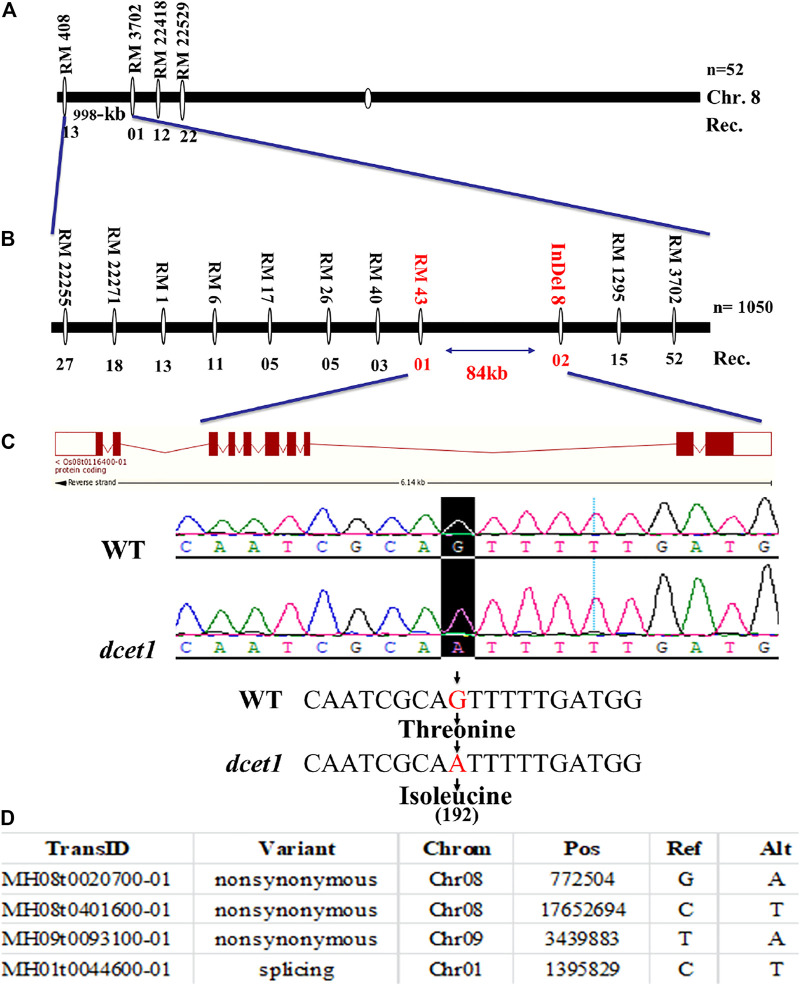
Representation of map-based cloning of the *DCET1* gene and MutMap candidate all index SNP genes. Primary mapping **(A)**. Fine mapping **(B)**. Structure of the *DCET1* gene and single-nucleotide substitution in mutant *dcet1* from G (threonine) to A (isoleucine) on 192 amino acids **(C)**. MutMap candidate All index SNP genes **(D)**.

First, a map-based cloning technique was used to identify the gene responsible for the *dcet1* mutant phenotype. The F_2_ population derived from the cross between the *dcet1* mutant and 02428 (sp. japonica) was used for genetic mapping. For linkage and primary mapping, we used 52 F_2_ plants and eventually mapped the *DCET1* gene at 998-kb intervals between SSR markers named RM408 and RM3702 on chromosome 8 (Figure 9A). The region was further narrowed down to 84 kb by using 1,050 F_2_ plants, between markers Indel8 and RM43 (Figure 9B), containing ten genes based on bioinformatics analysis (http://www.gramene.org/). All the ten potential candidate genes within this region were sequenced, and a single-bp substitution was identified from G (threonine-192) to A (isoleucine-192) in the fifth exon of the gene corresponding to LOC_Os08g02330 (http://rice.plantbiology.msu.edu/), also annotated as Os08t0116400-01 (http://rapdb.dna.affrc.go.jp/). This gene is predicted to be a CTC-interacting domain-11 (CID 11)–like gene**,** an RNA-binding protein having 10 exons, two domains named RRM1 and RRM2, and a C-terminal PAM2 conserved site (Figure 9C).

Second, the MutMap cloning approach was also used to re-confirm the LOC_Os08g02330 responsible for the *dcet1* mutant according to the previous description (Abe et al., 2012). For MutMap ,30 F_2_ *dcet1* mutant plants were used, and the results identified four ORFs annotated as MH08t0020700-01, MH08t0401600-01, MH09t0093100-01, and MH01t0044600-01 (http://rice.hzau.edu.cn/rice/), among which MH08t0020700-01 was lying in the abovementioned 84-kb fine-mapped region (Figure 9D; Supplementary Figures S3A,B). All the four ORFs were sequenced and aligned with the reference sequence. The mutation was found only in MH08t0020700-01, which was the same fine-mapped gene also annotated as Os08t0116400-01 (http://rapdb.dna.affrc.go.jp/).

### Phylogenetic Analysis of *DCET1*

**FIGURE 10:**
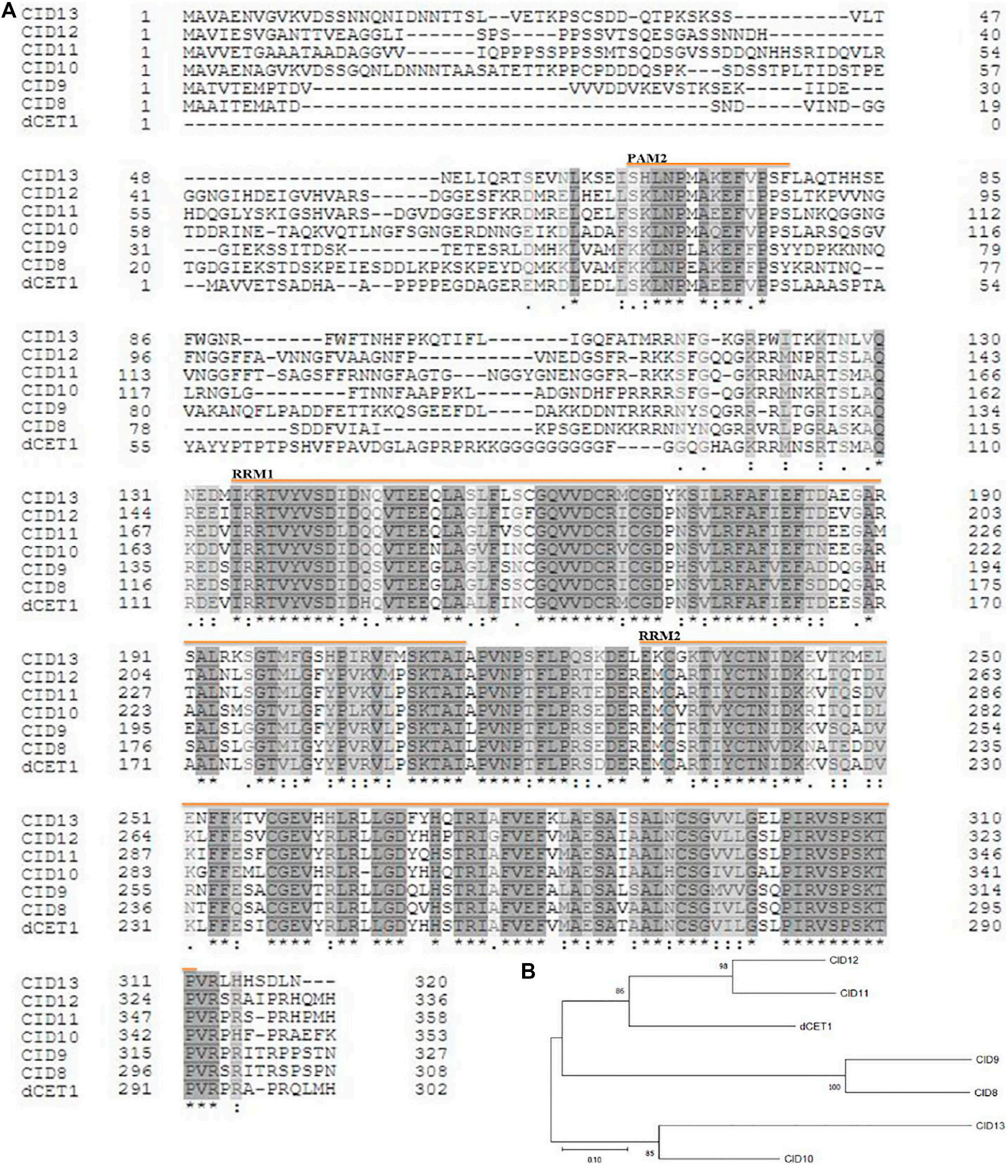
Representation of sequence alignment and maximum likelihood tree of *DCET1* and CID-D proteins. Sequence alignment of *DCET1* and CID-D proteins **(A)**. Black and star indicate amino acid identity across all proteins; Grey and double dot indicate identity in most proteins; Light black and single dot indicate identity in many proteins; Yellow line with name indicates the region of the specific domain. Phylogenetic tree of *DCET1* and selected CID-D proteins **(B)**.

Phylogenetic analysis showed that *DCET1* belongs to *Arabidopsis* CID11–like proteins having two RRM domains and a conserved motif at the C-terminus, designated as PAM2 (PABP-interacting motif 2), and are suggested to encode highly related RNA-binding proteins (RBPs). Previously in *Arabidopsis*, 13 CIDs were divided into four groups: group A consists of two genes CID1 and CID2, group B also contains two genes CID3 and CID4, group C consists of three genes CID5, CID6, and CID7, while group D includes six genes CID8, CID9, CID10, CID11, CID12, and CID13 (Bravo et al., 2005). Group D CIDs are the closest homologues of *DCET1*, holding the same two RRM domains and a basic C-terminal conserved region (Figures 10A,B), which can guide the protein interactions. The presence of RRMs and PAM2 in group D plus the previous study on CID12, one of the group D members, strongly suggests that in these proteins, there may be a dual-type of interaction taking place, that is, targeting mRNA and binding to PABP (Hecht et al., 1997). All the six members of this group are mainly uncharacterized proteins (with some exceptions for CID12, shown to express during early embryogenesis and growing organs) with unknown functions (Bravo et al., 2005). In order to get further insight of the evolutionary and functional conservation among *DCET1* and its relatives in various plants species, additional 17 amino acid sequences were used. The peptide alignment and its phylogenetic tree showed that, including *DCET1*, all the 17 proteins had high conservation for the active sites (RRM1, RRM2, and PAM2), inferring that these functional positions are evolutionarily conserved in land plants (Supplementary Figures S4A,B). Our results showed severe defects in the *dcet1* anther phenotype during pollen development; we propose that like *DCET1*, its homologues, including CIDs, may have the same functional defects.

### Expression Pattern of *DCET1*

**FIGURE 11:**
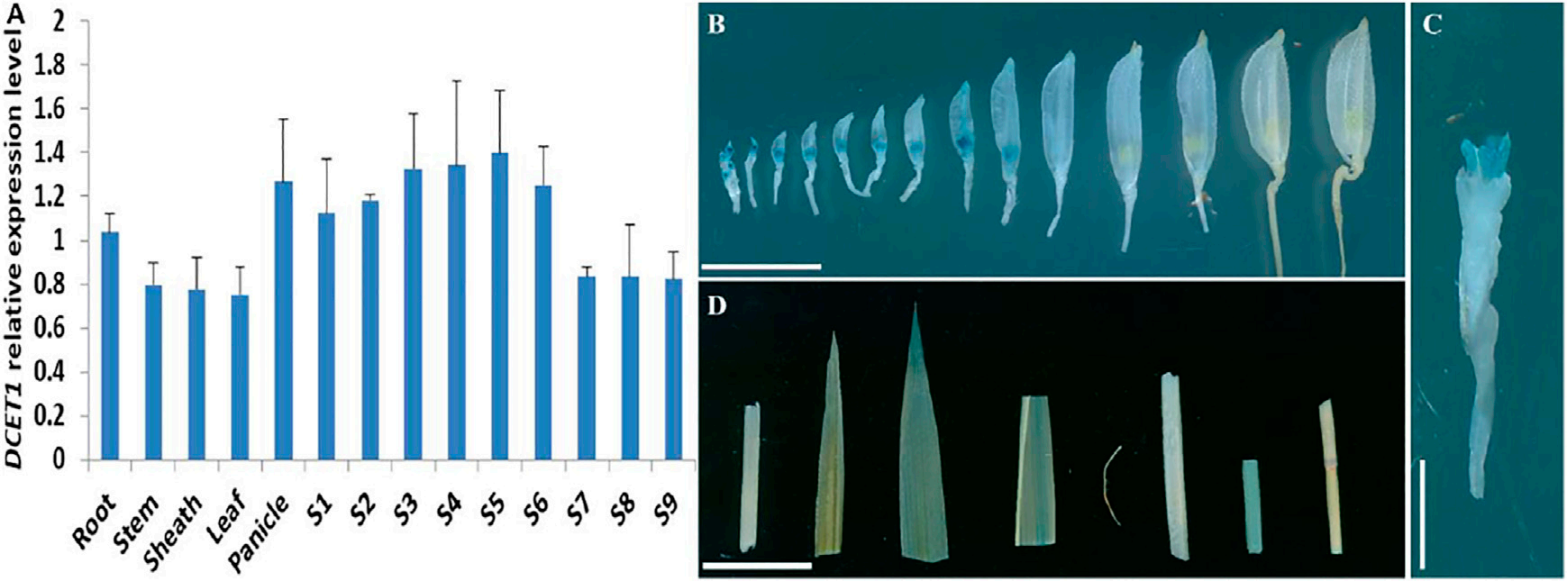
Expression analysis of *DCET1*. QPCR relative expression level at different developmental stages of spikelets (on length basis) and other plant tissues **(A)**. S1–S10 anthers dissected from florets with 2–3 mm, 3–4 mm, 4–5 mm, 5–6 mm, 6–7 mm, 7–8 mm, 8–9 mm, 9–10 mm, >10 mm; GUS-staining of the *proDCET1*:*GUS* for transgenic line spikelets at different anther stages (1–14) **(B)**. GUS staining of the *proDCET1*:*GUS* for transgenic florets with palea and lemma removed **(C)**. GUS staining of the *proDCET1*:*GUS* leaf, root, sheath, and stem **(D)**. Scale bars **(B–D)** = 20 µm.

Our early results suggested that mutation in *DCET1* led to abortive pollen in the *dcet1* mutant without affecting the vegetative growth, indicating that *DCET1* might be specifically expressed in anther. Thus, we performed the expression analysis through quantitative real-time-PCR (qRT-PCR) for different anther developmental stages and other WT plant tissues (Figure 11A). The level confirmed that although expressed in all tissues, the *DCET1* was ubiquitously expressed in anthers from stage 1 to 10 with the highest relative expression at stage 7 and 8 (Figure 11A). To further explicit the spatial and developmental nature of *DCET1* expression, we performed the native promoter-GUS analysis, which revealed that, although found to be expressed from stage 1 to 10 of anther development, the highest GUS staining expression was observed at stages 7 and 8 (Figures 11B,C). The other plant parts such as root, stem, sheath, and leaf showed negligible GUS staining (Figure 11D). These results were consistent with those of qRT-PCR, showing the highest relative expression levels in anthers from stages 7 to 8 (Figure 11A).

### Functional Validation of the *DCET1* Gene

**FIGURE 12:**
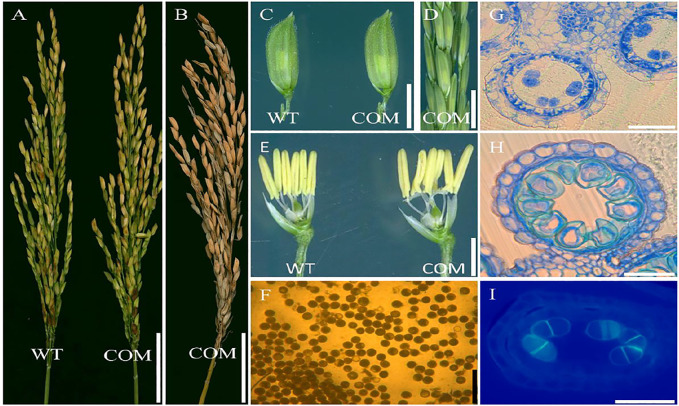
Phenotypic characterization of the complemented line (COM) of *DCET1* and wild-type (WT). Panicles of WT and COM line at physiological maturity **(A)**. COM line panicle at harvest maturity **(B)**. Spikelets of the WT and COM line **(C)**. COM line panicle at stage 13 **(D)**. Spikelets of WT and COM line after the removal of palea and lemma **(E)**. Pollen grains of COM line after staining with 1.2% I2-KI staining solution **(F)**. Anther section of the COM line at stage 8b and 10, respectively, **(G,H)**. Aniline blue staining of the anther section at stage 8b **(I)**. Scale bars = 1 cm in **(A,B)**, 2 mm in **(C,D)**, 1 mm in **(E)**, 100 µm in **(F),** and 15 µm in **(G–I)**.

To confirm the mutation in the LOC_Os08g02330 caused male sterile phenotype in *dcet1*, a complementary vector of an entire 10,249-bp genomic sequence, consisting of 6,263 bp whole locus of *DCET1* (LOC_Os08g02330), 2,943 bp upstream sequence, and 1,043 bp downstream sequence was transformed into homozygous *dcet1* plants. The complementary transgenic plants produced normal seed setting (Figures 12A,B) with dark yellow anthers (Figures 12C–E), and round black pollen grains in I_2_-KI staining (Figure 12F). Furthermore, anther transverse section analysis was also carried out in complementary lines for the critical abnormal stages in anther development. Sections studies of stages 8b and 10 clearly showed the normal fertile phenotype in complementary plants (Figures 12G,H), like WT as described in Figures 1B–E. As the most critical abnormalities in the *dcet1* mutant for callose wall was observed at the anther development stage 8b, the aniline blue staining was carried out for this stage in the complementary line, which showed the normal callose layer (Figure 12I) resembling WT as described in Figure 6H. These results confirmed that the single bp substitution in LOC_Os08g02330 was responsible for the sterile phenotype in the *dcet1* mutant.

To gain further insights into the role of LOC_Os08g02330 in rice male sterility, we also generated a *dcet1-2* transgenic mutant by targeted knockout through the CRISPR/Cas9 system. We found two homozygous transgenic plants with similar single bp mutations in the targeted region (Supplementary Figure S2E). We used these mutated transgenic plants for phenotypic analysis, which showed the same male reproductive system defects as previously observed in *dcet1* (Supplementary Figure S3A). These findings confirmed that the mutation in the Os08t0116400-01 gene, named *DCET1*, was responsible for the defects in male fertility in the *dcet1* mutant phenotype.

## Discussion

Rice is a monocot and has many resources for understanding and exploring the male reproductive system development (Uzair et al., 2020). Abortive pollen is paramount for hybrid rice breeding; several genetic and environmental factors are responsible for pollen sterility. In *Arabidopsis* and rice, many genes are reported for pollen sterility through altered biosynthesis and biochemical degradation of the callose layer (Verma and Hong, 2001; Shi et al., 2016), abnormal PCD-induced tapetum degradation (Sun et al., 2018; Uzair et al., 2020), and defective pollen wall (Dong et al., 2005; Lu et al., 2014; Zou et al., 2017b). Here, we have cloned and functionally characterized a male sterility-related gene, *DCET1*, having normal vegetative growth but a defective male reproductive system through the abnormal callose wall, defective pollen wall, and delayed tapetum degradation and hence a complete male-sterile phenotype. *DCET1* belongs to *Arabidopsis* CID-D–like proteins of mainly unknown functions, suggested to perform the dual-type of interactions, targeting mRNA and binding to PABP (Hecht et al., 1997; Bravo et al., 2005). It is proposed that *DCET1* plays an important role in regulating and modifying male sterility through complex biological processes in rice.

### *DCET1* is Required for Callose Biosynthesis and Degradation

Callose (β-1,3-glucan) is an essential component in higher plants and is synthesized by CalS and deposited at various stages during reproductive system development to various cell organelles within the microsporocytes and megaspores (Shi et al., 2014; Shi et al., 2016). The callose wall is a provisional cell wall responsible for normal permeability and releasing and preventing the fusion of sibling microspores and the plasmic membranes (Lu et al., 2014). During microsporogenesis, the meiocytes till the division of meiotic dyads and tetrads need to be properly surrounded and separated by the callose wall. The degeneration and dissolution of callose through the callase enzyme secreted by both the tapetum and meiocytes itself after tetrad formation is the precursor for sporopollenin depositions on the primexine surface (Ariizumi and Toriyama, 2011). During the tetrad stage, the callose wall provides protection to the primexine by fusion with the plasma membrane and outside the locule (Ariizumi and Toriyama, 2011). Hence, proper time and amount of callose accumulation and dissolution is necessary for pollen wall and fertility (Dong et al., 2005). Glucan synthase–like (GSL) is a complex and important family that is involved in callose deposition in microspore developments. An *Arabidopsis* AtGSL10 (CalS9) knock-out line is involved in pollen development and aberrant asymmetric microspore formation at the mitotic division stage (Huang et al., 2009). Previous studies revealed that mutation in the callose synthase CalS5 leads to defective exine patterning; specifically, the microsporocytes of *CalS5-1* and *CalS5-2* mutants are not only missing with perceptible callose wall but also in exine patterning (Nishikawa et al., 2005; Shi et al., 2014). In the present study, we reported that the callose accumulation in *dcet1* mutant meiocytes is negligible, while during the tetrad stage, the callose wall was not formed around the microspore, although some amount of callose was seen to spread over the surface of the microspore and locule as shown in Figure 6. Our results are consistent with previously reported phenomena in the rice *GSL5* mutant, which showed the defects in callose accumulation, leading to pollen exine formation defects (Shi et al., 2014). These findings confirmed that *DCET1* is required for proper callose synthesis, not only for proper callose wall formation but also for pollen exine patterning.

### *DCET1* is Needed for Tapetal PCD

Tapetum is one of the four somatic layers in rice anther, lying to the innermost side, and is directly attached to the anther locule where the formation, division, and growth of microspores take place (Ariizumi and Toriyama, 2011). The previous study shows that the tapetum provides not only nutrients, metabolites, and sporopollenin precursors but also the materials for exine and tryphine formation; hence, any mutation in tapetum-related genes can lead to deformed tapetum and exine morphologies (Liu et al., 2017; Zhu et al., 2017). Likewise, the proper time and amount of tapetal cell PCD are considered necessary for callose degradation, exine wall patterning, and pollen viability through secretion of callases and sporopollenin precursors (Dong et al., 2005).

*dcet1* exhibits delayed tapetal cell degradation and abnormal decay for other outer somatic layers validated by semi-thin section observation, TEM analysis, and TUNEL assay (Figures 2, 4, 6). *dcet1* showed the increase in somatic layer PCD specifically for tapetal cells till the pollen maturation, which confirmed the delayed and abnormal nature of tapetal cell PCD and outer anther layer degradation in the *dcet1* mutant. Furthermore, the semi-thin studies and TEM analysis showed normal tapetum till the meiosis initiation stage. Interestingly, the tapetum was not degraded properly until the microspore maturation stage compared with the WT plants (Figures 2, 4). These results are consistent with *OsGPAT3*, and *ptc2* mutants as previously characterized (Sun et al., 2018; Uzair et al., 2020). Our results indicate that *DCET1* plays a critical role in regulating PCD-induced tapetal cell degradation; however, the proper mechanism and pathway are still unclear and need further elucidation for male sterility functions in rice crops.

### Proper Callose; Timely Tapetal PCD; or Both; Needed for Exine in *dcet1* Mutant

It is a well-known fact that both the tapetum and the developing microspores contribute to exine formation, and any interference in these processes can lead to the male sterility phenotype (Piffanelli et al., 1998; Yang et al., 2017). During microsporogenesis, the callose is synthesized and accumulated on microsporocytes, tetrads, and microspores (Dong et al., 2005). At the tetrad stage, the tapetum and meiocytes also secrete callases for callose degradation, but before the callases degrade the callose, primexine appears on the microspore surface. At the same time, the tapetum also starts to release sporopollenin precursors to be deposited on the primexine surface, responsible for the final exine wall and pollen fertility (Men et al., 2017). Furthermore, the normal tapetal PCD is considered essential for normal exine patterning (Lu et al., 2014). Here, the deposition of the sporopollenin precursor on the primexine surface needs the presence of a proper callose layer to guarantee the process by preventing cell fusion and cohesion. As mentioned, primexine is important for exine patterning, but for proper primexine formation, both the callose and tapetum are necessary. In rice and *Arabidopsis*, several genes and transcription factors have been identified for callose regulation, tapetal PCD, and pollen wall development and patterning. Based on their functional relations, we divided these genes and transcription factors into three groups. The callose-regulated genes which can affect the pollen wall are *CalS5* (Dong et al., 2005), *GSL5* (Shi et al., 2014), and *CDM1* (Lu et al., 2014). Those which affect tapetal PCD and also the pollen wall are *PTC1* (Li et al., 2011), *PTC2* (Uzair et al., 2020), *OsCOS12* (Yang et al., 2017), *TIP3* (Yang et al., 2019b), *OsGPAT3* (Sun et al., 2018), and *EAT1* (Niu et al., 2013). There are also several genes and transcriptional factors which are not only associated with the callose layer and tapetum PCD but also with the pollen wall such as *AtMYB103* (Zhang et al., 2007) and *OsDEX1* (Yu et al., 2016).

Several studies have confirmed that alteration in sporopollenin constituents are highly related to exine patterning, as both of the cutin and sporopollenin share common monomers (Liu et al., 2017). So, the changes in cutin monomers can lead to the sporopollenin precursor deviations and ultimately to pollen sterility. As reported for *DPW* to produce the critically needed C16:0 fatty alcohols for exine development, the *dpw* mutant has reduced sporopollenin deposition on the primexine surface of the pollen grains and hence having degenerated pollen grains with irregular exine and abnormal cuticle (Shi et al., 2011). Wax-defective anther1 (*WDA1*) is associated with the formation of cuticle and wax deposition by the biosynthesis of very long-chain fatty acids (VLCFA) in rice, and its mutant (*wda1*) shows abnormal anther wall and pollen exine formation (Jung et al., 2006). The *dcet1* mutant is deficient in cutin monomers, while the wax constituents were mainly over-accumulated like both of *ptc2* and *dpw* phenotypes in rice (Shi et al., 2011; Uzair et al., 2020) (Figures 8B–D). The maize *apv1* mutant has shows the same cuticular lipid trend as that of *dcet1*, and having a complete male sterile phenotype (Somaratne et al., 2017). Specifically, the delayed tapetal PCD phenotype of *dcet1* resembled that of the *ptc2* mutant; both expressed almost the same anther and pollen surface patterns in SEM and lipidic analysis (Uzair et al., 2020) (Figures 3A–J, 8A–D). From the previous literature, it can also be assumed that the defects of pollen exine in *dcet1* may reflect that *DCET1* disturbs the mutual biosynthetic pathway for the two biopolymer’s synthesis, the sporopollenin and cutin. Still for exploring the meticulous pathway, responsible for the dcet1 phenotype, further studies are needed with all related genes.

As previously reported in the *gls5* mutant (Shi et al., 2014) in rice, the timing of callose deposition and degradation is important for exine wall formation. Here, we add the proper tapetal PCD, with the quoted sentence important for exine wall formation in the *dcet1* mutant. As we can assume from our results that either callose or tapetum or both could be the causative agents for exine malformation, hence further research and investigations are needed on the *DCET1* gene to solve the complexity.

In short, we have identified RNA recognition motifs containing protein and *DCET1*, which is required for pollen development by regulating callose biosynthesis, tapetal PCD, and pollen wall patterning. In *dcet1*, both callose biosynthesis and its degradation and tapetal cell PCD are abnormal, and eventually, the exine with its patterning is improper.

## Conclusion

The current study presents the functional characterization of the *DCET1* gene in rice. Our results showed that *DCET1*, belonging to RNA recognition motif (RRM)–containing proteins, is potentially involved in male sterility in rice. In brief, *DCET1* regulates callose biosynthesis and degradation and pollen exine formation by affecting exine wall patterning, including abnormal nexine, collapsed bacula, irregular tectum, and timely PCD by delaying the tapetal cell degeneration. As a result, the microspore of *dcet1* was swollen and abnormally bursted and even collapsed within the anther locule with complete sterility. Taken together, the disruption of *DCET1* function leads to the extension of male sterility and reproductive system in rice and will play an important role in hybrid rice breeding programs. CID-like family proteins are the closest homologs of *Arabidopsis*; however, many have unknown functions (except CID12 expressed during embryogenesis). Therefore, *DCET1* receives key importance and provides novel insights for the prediction and characterization of RBDs, in general, and CIDs in specific in all eukaryotes.

## Data Availability

The original contributions presented in the study are included in the article/Supplementary Material; further inquiries can be directed to the corresponding author.
