# Supplementary material for: DCET1 Controls Male Sterility Through Callose Regulation, Exine Formation, and Tapetal Programmed Cell Death in Rice
Source: Front Genet. 2021 Nov 24;12:790789. doi: 10.3389/fgene.2021.790789 (PMC8652220; doi:10.3389/fgene.2021.790789)
Supplement: Supplementary file 1 [file Table1.docx]

**Supplimentry Data**

Table S1. List of all primer names and corresponding sequences used for mapping, qRT and vector construction etc.

| **Primer name** | **Genetic Mapping** | | |
| --- | --- | --- | --- |
|  | **F** | | **R** |
| RM408 | AATTGCCCAACGAGCTAACTTCC | | TGAGCTGTTTGTGCTCTTCTACTTCG |
| RM3702 | CCAGCACCATCTGAAATAGAAGC | | CCATTGATTAGGACGGTCAAAGG |
| RM22418 | GCAGTATCACGCAGTAGCACACC | | CCATCCTCTTCCTCATCACACG |
| RM22529 | TGCGAGTATTTAACTCACCCATCC | | CTTGCCTCACAAGATCCAAACC |
| RM1295 | GTGGAAGTTGAACGGCAACTCG | | AGAATCCGCTTCTGGAAGTCTCG |
| Indel 8 | TTCAAAAACAAATCGTGGGGC | | TTCGGGTCTCACCTACCCT |
| RM43 | ATCTTGCATGTAGGATGAGCAA | | AGGATGAAGCTTTTGCTTCCC |
| RM40 | TCGTGCGCTACTCTCTTTTA | | GTGGAGGCCTTCATGTTCTAT |
| RM26 | ATATATCGCGTACAGCCGTT | | GCACGATGTGTACTGGTCAT |
| RM17 | ACGGGTAGACATCCATCCTA | | TTAGTTTTGCCACCCACCC |
| RM6 | ATTTCCGGCATTTTGACAGG | | TGCTACAACTAATCATGCTGG |
| RM1 | GCGCTTCCTTGAACATCATT | | CGGATTGCATCCAAGAATCG |
| RM22271 | CAGTGCATCAATAGCGTAGATTGG | | AACTCGTAACGACTCCACAGTGC |
| RM22255 | AATTCTAGCAATGGCTCCTCTCG | | GACGAAGTCGATCAGCATCTCC |
| **qRT and UBIQUITIN** | | | |
| qRT | GTGGACTGTCGTATGTGTGG | | CAGTTTTTGATGGCAGCACC |
| UBQ | GCTCCGTGGCGGTATCAT | | CGGCAGTTGACAGCCCTAG |
| **GUS** | | | |
| *DCET1*-G | CGGTACCCGGGGATCCAAAAACGCCAGCTTTGGTCT | | CTCAGATCTACCATGGCCCCGCAGCAACGGGAA |
| **Complementation** | | | |
| *DCET1*-C | CCATGATTACGAATTCGTGTACACCAAGCCAGACAA | | TACCGAGCTCGAATTCCCAAACGATGGAGACCAGAG |
| **GFP** | | | |
| *DCET1*-L | TAGAACTAGTGGATCCATGGCCGTCGTCGAGAC | GCTTGATATCGAATTCGTGCATCAGCTGGCGAG | |
| **MutMap predicted genes primers** | | | |
| MH01t004460 | AGGATGAAGTCTTTCCTGCAA | AGCTGCATTTCCTGTTTCCA | |
| MH09t009310 | GCACTGTTATTGGAGGCAGA | TCTCTGTGGTCCAAGTGTTC | |
| MH08t0020700/ *DCET1* | TTTTCTGGCTCCACACTTGC | GTCCTTGCACACATCTCACG | |
| MH08t0401600 | TCAAATCCGGTTGTTCCGAG | ACACTCCTCCTTACCCCAC | |
| **CRISPR/Cas9, Target and confirmatory primers** | | | |
| Target | tcatccggcgcttcccggcgtgg |  | |
| R8015-441+ | ATCTGGAGGACCTGCTCTCC |  | |
| R8015-1128- | GCGTACTGTACATCCCAACC |  | |
