# Supplementary material for: DCET1 Controls Male Sterility Through Callose Regulation, Exine Formation, and Tapetal Programmed Cell Death in Rice
Source: Front Genet. 2021 Nov 24;12:790789. doi: 10.3389/fgene.2021.790789 (PMC8652220; doi:10.3389/fgene.2021.790789)
Supplement: Supplementary file 2 [file DataSheet1.pdf]

FIG. S1. Represents 4,6-diamidino-phenylindole (DAPI)-stained chromosome of male meiosis in WT and *dcet1* mutant. (**A-H**, **I-L**) Showing WT chromosomes; (**E-H, M-P**) Showing *dcet1* chromosomes. (**A**, **E**) Prophase I; (**B**, **F**, **C**, **G**) Metaphase I; (**D**, **H**) Anaphase I; (**I**, **M**) Telophase I; (**J**, **N**) Metaphase II; (**K**, **O**) Dyad; (**L**, **P**) Tetrad. Scale bars = 5 mm.

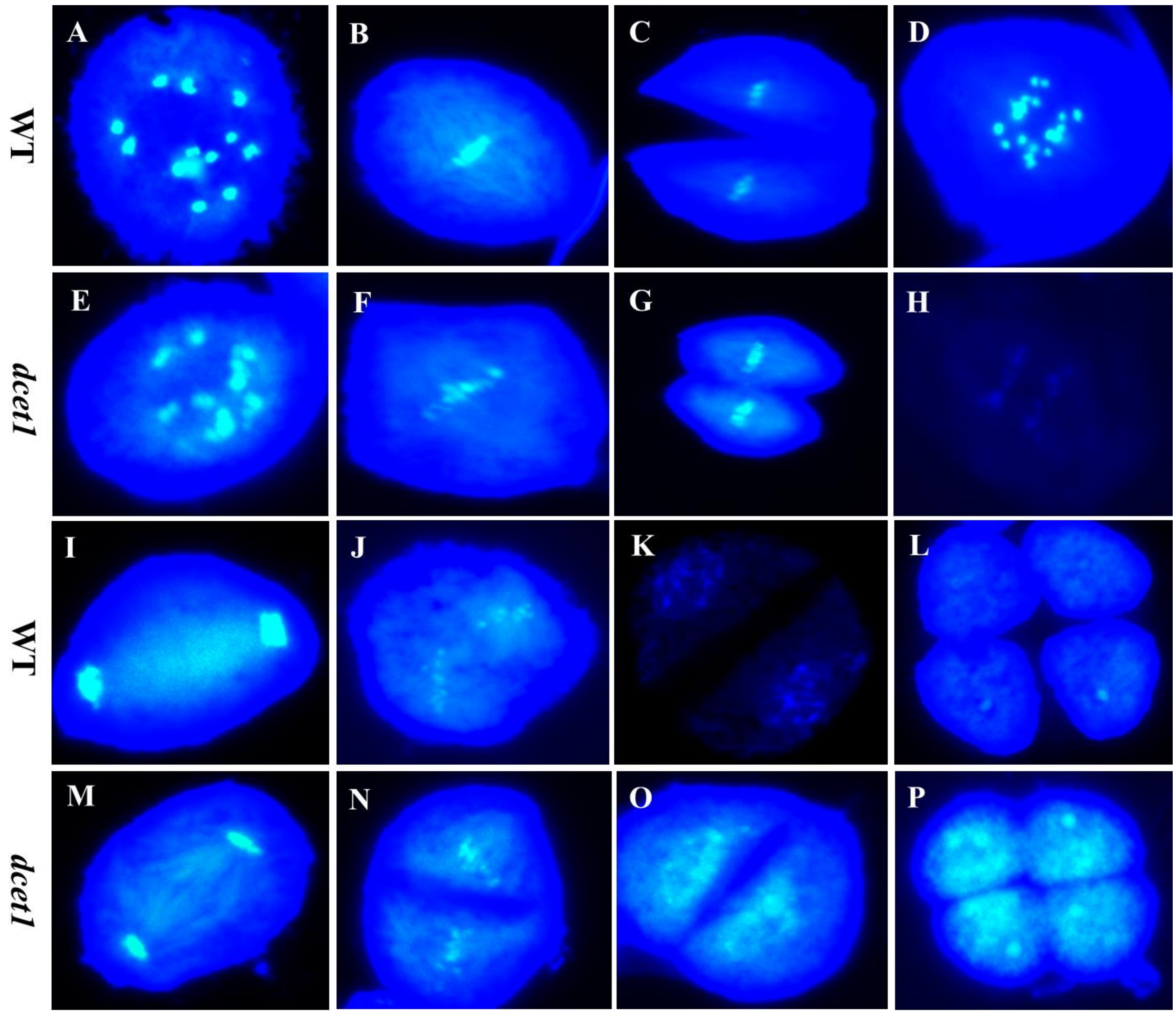

FIG. S2. Representation of the phenotypic characterization of CRISPR/Cas9-mediated (*DCET1-2*) plants in comparison to wild-type (WT). Spikelets of WT and *dcet1-2* after the removal of palea and lemma (**A**); Anther of WT and *dcet1-2* (**B**); Semi-section of *dcet1-2* mature anther(**C**); Pollen grains of *dcet1-2* after staining with 1.2% I2-KI staining solution (**D**); Target region alignment of forward (*dcet1*-2F) and reverse (*dcet1-2*R) sequences with reference gene (Ref.) shows single nucleotide (C) deletion in *dcet1-2* (**E**). Scale bars= 2 mm in (**A**, **B**), 15 µm in (**C**) and 100 µm in (**D**).

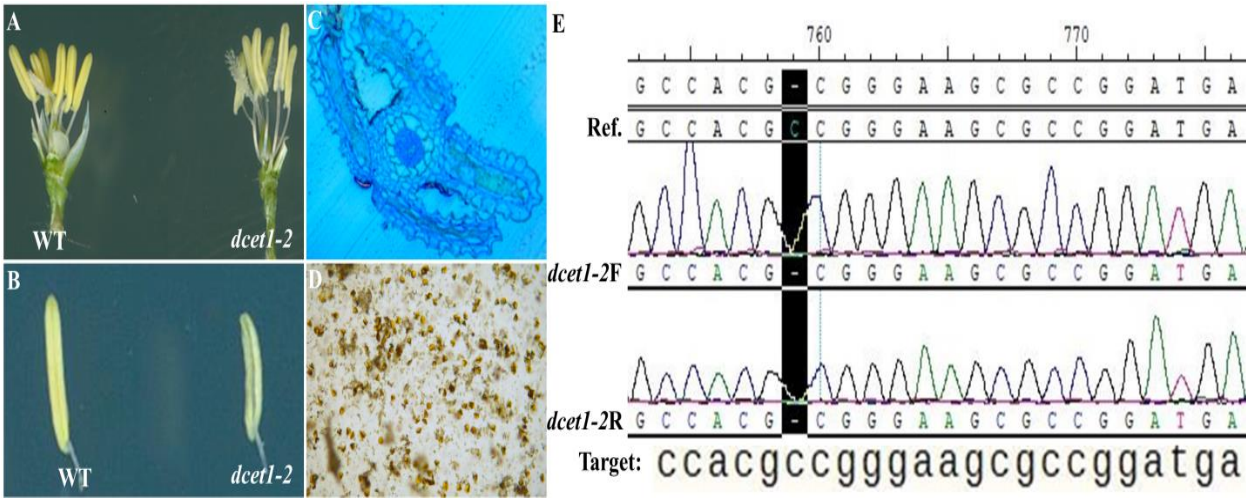

FIG. S3. Represents MutMap F_2 SNP index (**A**); and SNP index manhattan analysis (**B**). The mutated chromosome 08 is encircled by red line.

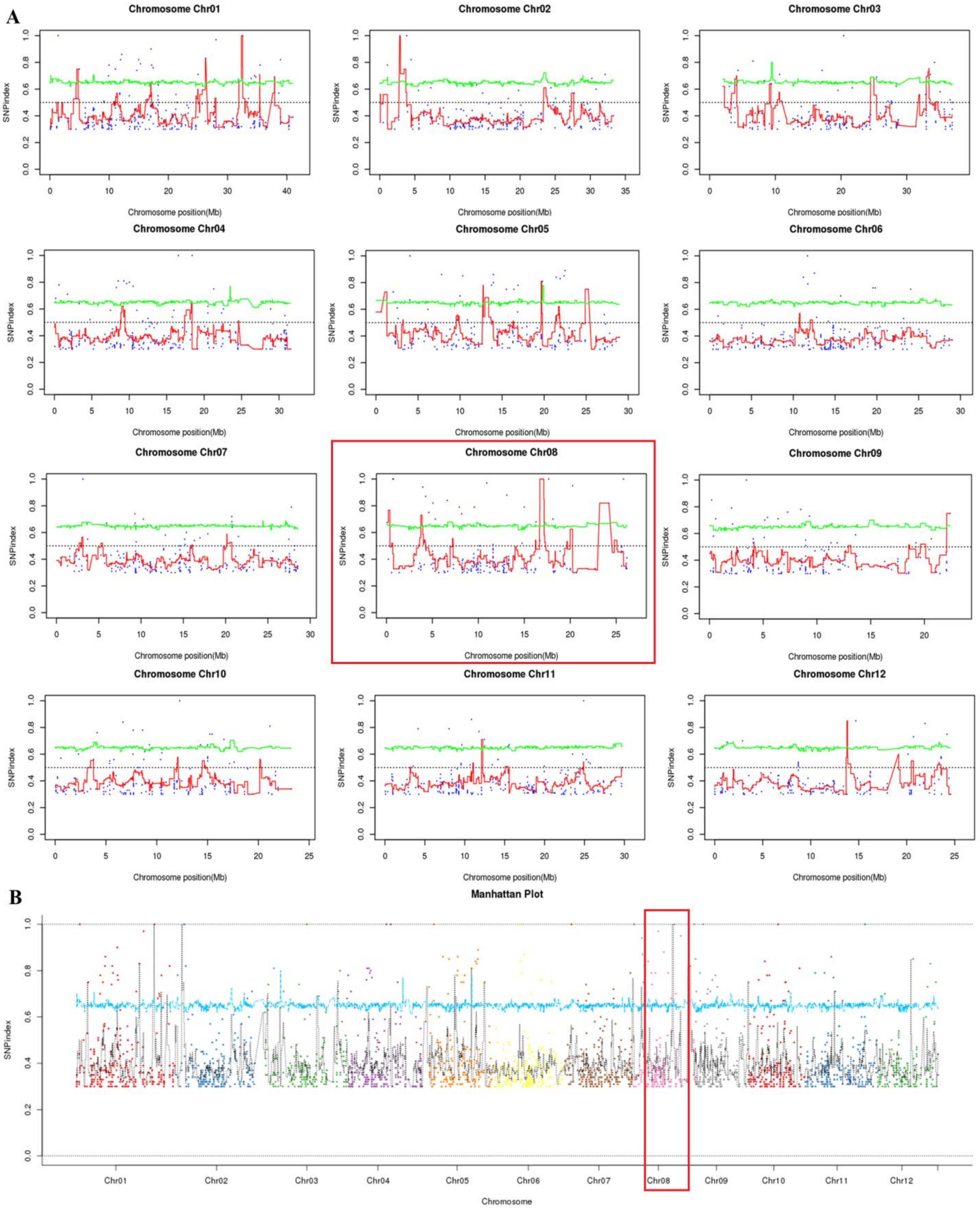

FIG. S4. Represents sequence alignment (**A**), and phylogenetic tree of *DCET1* and 17 amino acids (**B**). The evolutionary history was inferred using the Neighbor-Joining method (Saitou and Nei, 1987). Sum of branch length = 0.58659585; bootstrap = 1000 replicates.

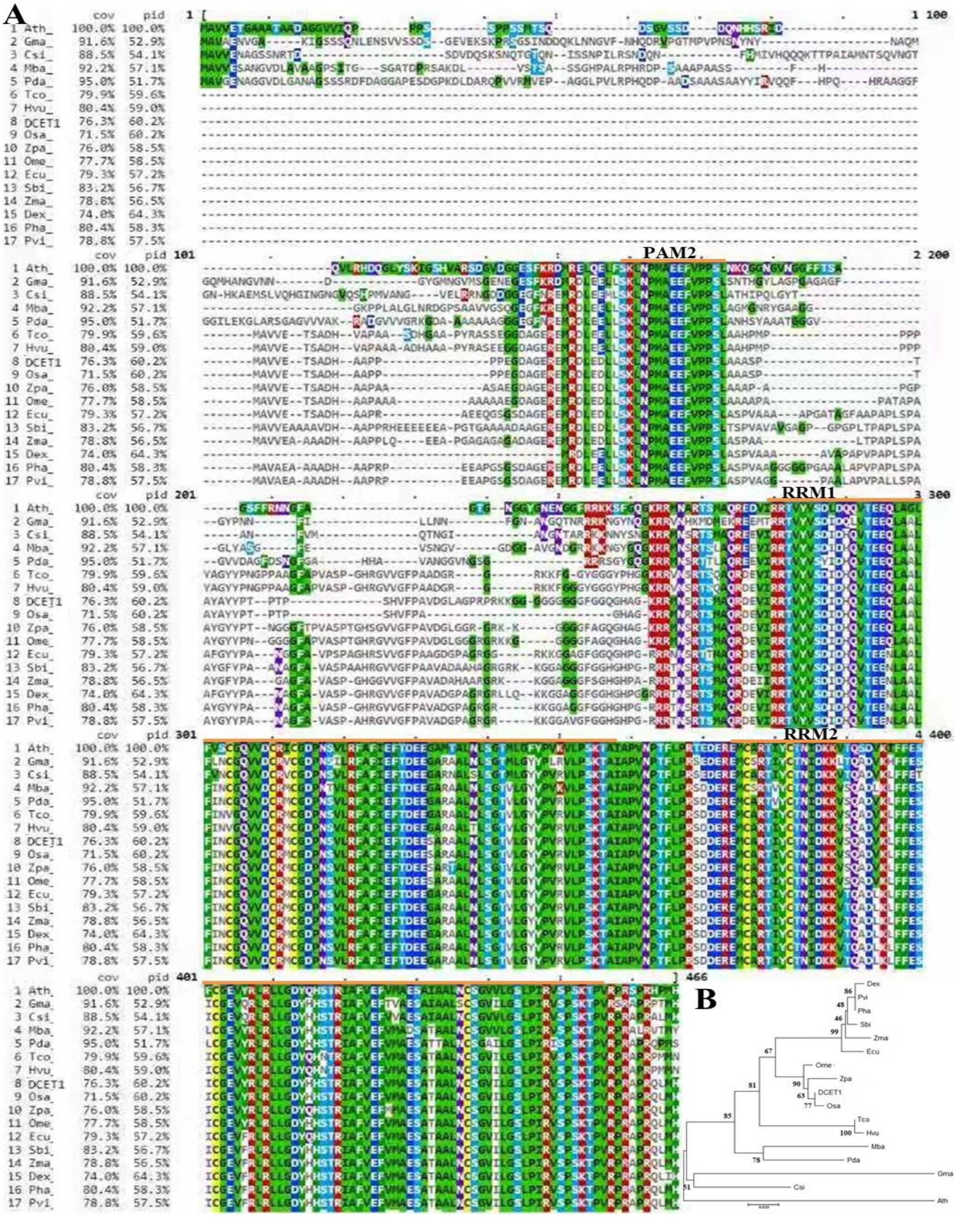
A
B
PAM2
RRM1
RRM2
cov
pid
Ath
Gma
Csi
Mba
Pda
Tco
Hvu
DCET1
Osa
Zpa
Ome
Ecu
Sbi
Zma
Dex
Pha
Pvi
